# Supplementary material for: Expanding plant genome-editing scope by an engineered iSpyMacCas9 system that targets A-rich PAM sequences
Source: Plant Commun. 2020 Jul 22;2(2):100101. doi: 10.1016/j.xplc.2020.100101 (PMC8060698; doi:10.1016/j.xplc.2020.100101)
Supplement: Document S1. Supplemental Figures 1–4 and Supplemental Tables 1 and 2 [file mmc1.pdf]

Plant Communications, Volume *1*

Supplemental Information

Expanding Plant Genome-Editing Scope by an Engineered iSpyMac-Cas9 System That Targets A-Rich PAM Sequences

Simon Sretenovic, Desuo Yin, Adam Levav, Jeremy D. Selengut, Stephen M. Mount, and Yiping Qi

Expanding Plant Genome Editing Scope by An Engineered iSpyMacCas9 System Targeting the A-rich PAM Sequences

Simon Sretenovic[1, ƚ], Desuo Yin[1,2, ƚ], Adam Levav[1,3], Jeremy D. Selengut[4], Stephen M. Mount[5], Yiping Qi[1,6, *]

[1]Department of Plant Science and Landscape Architecture, University of Maryland, College Park, Maryland 20742, USA; [2]Hubei Academy of Agricultural Sciences, Wuhan 430064, China; [3]Montgomery Blair High School, Silver Spring, Maryland 20901, USA; [4]Center for Bioinformatics and Computational Biology, University of Maryland, College Park, Maryland 20742, USA; [5]Department of Cell Biology and Molecular Genetics, University of Maryland, College Park, Maryland 20742, USA; [6]Institute for Bioscience and Biotechnology Research, University of Maryland, Rockville, Maryland 20850, USA.

ƚ These authors contributed equally to this work.

***Corresponding author**

Yiping Qi, Department of Plant Science and Landscape Architecture, University of Maryland, College Park, MD 20742, USA; Email: Yiping@umd.edu

SUPPLEMENTAL INFORMATION

- Supplemental Table 1. Oligos used in this study.
- Supplemental Table 2. T-DNA expression constructs used in this study.
- Supplemental Figure 1. Raw RFLP data of testing 4 (i)SpyMacCas9 variants at 4 target sites in 3 replicates.
- Supplemental Figure 2: Raw RFLP data of testing z-iSpyMacCas9 at 28 target sites in 3 replicates.
- Supplemental Figure 3. Mutated T0 lines induced by SpyMacCas9 and iSpyMacCas9 with OsPDS-sgRNA11.
- Supplemental Figure 4. Indel mutants generated by PmCDA1 based iSpyMacCas9 CBE with OsROC5-sgRNA05.

Supplemental Table 1. Oligos used in this study.

Name	Sequence 5'-3'	Description
AAAA-PDS-gR1-F	tggcATGAGTAGGAAGTTGTCCCGC	Forward primer to prepare gRNA
AAAA-PDS-gR1-R	aaacGCGGGACAACTTCCTACTCAT	Reverse primer to prepare gRNA
AAAA-ROC5-gR1-F	GTGTgctccagttgcgtcttggt	Forward primer to prepare gRNA
AAAA-ROC5-gR1-R	AAACaccaagacgcaactggagc	Reverse primer to prepare gRNA
AAAT-PDS-gR2-F	TGGCatagcacaacgagaaccttaa	Forward primer to prepare gRNA
AAAT-PDS-gR2-R	AAACttaaggttctcgttgtgctat	Reverse primer to prepare gRNA
AAAT-ROC5-gR2-F	TGGCagctgcttacaaaaccggag	Forward primer to prepare gRNA
AAAT-ROC5-gR2-R	AAACctccggttttgtaagcagct	Reverse primer to prepare gRNA
AAAG-PDS-gR3-F	tggcAGTAGGAAGTTGTCCCGCA	Forward primer to prepare gRNA
AAAG-PDS-gR3-R	aaacTGCGGGACAACTTCCTACT	Reverse primer to prepare gRNA
AAAG-ROC5-gR3-F	GTGTgcaagccaacgcctggcgcc	Forward primer to prepare gRNA
AAAG-ROC5-gR3-R	AAACggcgccaggcgttggcttgc	Reverse primer to prepare gRNA
AAAC-PDS-gR4-F	tggcAGGTCTTGGAAAGTCCTGGC	Forward primer to prepare gRNA
AAAC-PDS-gR4-R	aaacGCCAGGACTTTCCAAGACCT	Reverse primer to prepare gRNA
AAAC-ROC5-gR4-F	GTGTgctccagttgcgtcttggta	Forward primer to prepare gRNA
AAAC-ROC5-gR4-R	AAACtaccaagacgcaactggagc	Reverse primer to prepare gRNA
TAAA-PDS-gR5-F	tggcAAATCTCTTGTCTTAAGGAA	Forward primer to prepare gRNA
TAAA-PDS-gR5-R	aaacTTCCTTAAGACAAGAGATTT	Reverse primer to prepare gRNA
TAAA-ROC5-gR5-F	GTGTgccgctccagttgcgtcttgg	Forward primer to prepare gRNA
TAAA-ROC5-gR5-R	AAACccaagacgcaactggagcggc	Reverse primer to prepare gRNA
TAAT-PDS-gR6-F	GTGTgtcatgtgtgttgcatatgag	Forward primer to prepare gRNA
TAAT-PDS-gR6-R	AAACctcatatgcaacacacatgac	Reverse primer to prepare gRNA
TAAT-ROC5-gR6-F	TGGCatgcatcaaatgcaacacg	Forward primer to prepare gRNA
TAAT-ROC5-gR6-R	AAACcgtgttgcatttgatgcat	Reverse primer to prepare gRNA
TAAG-PDS-gR7-F	gtgtGGAGTGAAATCTCTTGTCT	Forward primer to prepare gRNA
TAAG-PDS-gR7-R	aaacAGACAAGAGATTTCACTCC	Reverse primer to prepare gRNA
TAAG-ROC5-gR7-F	GTGTgttacatttctccggttttg	Forward primer to prepare gRNA
TAAG-ROC5-gR7-R	AAACcaaaaccggagaaatgtaac	Reverse primer to prepare gRNA
TAAC-PDS-gR8-F	tggcATGGTCAACAATAGGCATGCA	Forward primer to prepare gRNA
TAAC-PDS-gR8-R	aaacTGCATGCCTATTGTTGACCAT	Reverse primer to prepare gRNA
TAAC-ROC5-gR8-F	TGGCActctgtgaaatgaatgcaag	Forward primer to prepare gRNA
TAAC-ROC5-gR8-R	AAACcttgcattcatttcacagagT	Reverse primer to prepare gRNA
GAAA-PDS-gR9-F	gtgtGTCCTGGCAAACAACctgca	Forward primer to prepare gRNA
GAAA-PDS-gR9-R	AAACtgcagGTTGTTTGCCAGGAC	Reverse primer to prepare gRNA
GAAA-ROC5-gR9-F	TGGCatgtcagaccaccgtctctgt	Forward primer to prepare gRNA
GAAA-ROC5-gR9-R	AAACacagagacggtggtctgacat	Reverse primer to prepare gRNA
GAAT-PDS-gR10-F	gtgtGTGAAATCTCTTGTCTTAAG	Forward primer to prepare gRNA
GAAT-PDS-gR10-R	aaacCTTAAGACAAGAGATTTCAC	Reverse primer to prepare gRNA
GAAT-ROC5-gR10-F	TGGCacctgtgggtgccgttcca	Forward primer to prepare gRNA
GAAT-ROC5-gR10-R	AAACtggaacggcacccacaggt	Reverse primer to prepare gRNA
GAAG-PDS-gR11-F	gtgtGCCTGAAAAGTCTGCAGTCT	Forward primer to prepare gRNA
GAAG-PDS-gR11-R	aaacAGACTGCAGACTTTTCAGGC	Reverse primer to prepare gRNA
GAAG-ROC5-gR11-F	GTGTgcttgtcgttctcctgcttga	Forward primer to prepare gRNA
GAAG-ROC5-gR11-R	AAACtcaagcaggagaacgacaagc	Reverse primer to prepare gRNA
GAAC-PDS-gR12-F	GTGTgtaaggtactactccggaaa	Forward primer to prepare gRNA
GAAC-PDS-gR12-R	AAACtttccggagtagtaccttac	Reverse primer to prepare gRNA

GAAC-ROC5-gR12-F	TGGCacgcaactggagcggcacga	Forward primer to prepare gRNA
GAAC-ROC5-gR12-R	AAACtcgtgccgctccagttgcgt	Reverse primer to prepare gRNA
CAAA-PDS-gR13-F	tggcATGAGTAGGAAGTTGTCCCG	Forward primer to prepare gRNA
CAAA-PDS-gR13-R	aaacCGGGACAACTTCCTACTCAT	Reverse primer to prepare gRNA
CAAA-ROC5-gR13-F	GTGTgcaagccaacgcctggcgc	Forward primer to prepare gRNA
CAAA-ROC5-gR13-R	AAACgcgccaggcgttggcttgc	Reverse primer to prepare gRNA
CAAT-PDS-gR14-F	TGGCacCTGAAGAAATCGGTTTAAAG	Forward primer to prepare gRNA
CAAT-PDS-gR14-R	aaacCTTTAAACCGATTTCTTCAGgt	Reverse primer to prepare gRNA
CAAT-ROC5-gR14-F	TGGCActcaagcactgttgccttag	Forward primer to prepare gRNA
CAAT-ROC5-gR14-R	AAACctaaggcaacagtgcttgagT	Reverse primer to prepare gRNA
CAAG-PDS-gR15-F	tggcaTTTTCCTTTATTCCTTAAGA	Forward primer to prepare gRNA
CAAG-PDS-gR15-R	aaacTCTTAAGGAATAAAGGAAAAt	Reverse primer to prepare gRNA
CAAG-ROC5-gR15-F	GTGTgcggcacgagaacgcgcttct	Forward primer to prepare gRNA
CAAG-ROC5-gR15-R	AAACagaagcgcgttctcgtgccgc	Reverse primer to prepare gRNA
CAAC-PDS-gR16-F	tggcAAGCAAGATCTTTTGCGGGA	Forward primer to prepare gRNA
CAAC-PDS-gR16-R	aaacTCCCGCAAAAGATCTTGCTT	Reverse primer to prepare gRNA
CAAC-ROC5-gR16-F	TGGCatcttcgttttaccaagacg	Forward primer to prepare gRNA
CAAC-ROC5-gR16-R	AAACcgtcttggtaaaacgaagat	Reverse primer to prepare gRNA
SM-21-RFLP-F3	cggcatcagctctcagtcac	For testing genome editing activity at SM21 site
SM-21-RFLP-R3	gatgcacacttctctcacg	For testing genome editing activity at SM21 site
SM-15-RFLP-F	CTCCAGCTTCTTGAAATAAG	For testing genome editing activity at SM15 site
SM-15-RFLP-R	GAGTAGTACCTTACGTATTA	For testing genome editing activity at SM15 site
SM-13-RFLP-F	GACCACGATGTGACTGCTATC	For testing genome editing activity at SM13 and SM17 sites
SM-13-RFLP-R	GAGTTGCTTCAGCATGGATAC	For testing genome editing activity at SM13 and SM17 sites
SM1-RFLP-F	GCTCCAGCAATCACGACCTG	For testing genome editing activity at SM1, SM25, SM31, and SM5 sites
SM1-RFLP-R	CTGATACAGTGATACTTGGC	For testing genome editing activity at SM1, SM25, SM31, and SM5 sites
SM23-RFLP-F	GAGTTCCATCAGTAAGTGC	For testing genome editing activity at SM23, SM11, and SM27 sites
SM23-RFLP-R	GTGTTCCTGATCGAGTGAAC	For testing genome editing activity at SM23, SM11, and SM27 sites
SM3-RFLP-F	GACTATGTATGAACAGGAG	For testing genome editing activity at SM3 site
SM3-RFLP-R	GCTCATATTCACTGTTAGTAG	For testing genome editing activity at SM3 site
SM32-RFLP-F	CCGTCTTGTGTAGGCTGTTC	For testing genome editing activity at SM32, SM10, SM8, SM2, SM24, SM30, and SM22 sites
SM32-RFLP-R	GTTGAGCTCGTCCTTGAGG	For testing genome editing activity at SM32, SM10, SM8, SM2, SM24, SM30, and SM22 sites
SM16-RFLP-F	CGAACTTGTCAAGATGGCAC	For testing genome editing activity at SM16, SM18, SM28 sites
SM16-RFLP-R	CTACGTCTTACACTATGTAAG	For testing genome editing activity at SM16, SM18, SM28 sites
SM14-RFLP-F	GTGGTCTGACATGTTCTCG	For testing genome editing activity at SM14, SM4, SM20 sites
SM14-RFLP-R	CATAGTGAACCATTATTGTG	For testing genome editing activity at SM14, SM4, SM20 sites
SM6-RFLP-F	GTCAACAGTATAGGTTCAC	For testing genome editing activity at SM6, SM26 sites
SM6-RFLP-R	CACAGTTGATTCCGCAAATTG	For testing genome editing activity at SM6, SM26 sites
SM12-RFLP-F	GTCCTCTGCTACAGTGACAG	For testing genome editing activity at SM12 site
SM12-RFLP-R	CTCCATTGCTGAGGATGTC	For testing genome editing activity at SM12 site
ISM-Ins_F	gtactcgatcggcCTCGCTATTGGGACTAACTC	For preparing pYPQ265H, 266B, and 262m3

ISM-Ins_R	ccgctggccgcttGTCCTCTCCTATTTTTGAGAG	For preparing pYPQ265H, 266B, and 262m3
zISMBE-BB_F	ataggagaggacAAGCGGCCAGCGGCGACGAA	For preparing pYPQ265H, 266B, and 262m3
zISMBE-BB_R	cccaatagcgagGCCGATCGAGTACTTCTTGTCAGCAGCTG	For preparing pYPQ265H, 266B, and 262m3
SM17-NGS-F1	ACAGTGGCTCCAGCAATCACGACCTG	NGS primer for Base Editing with z-iSpyMacas9 BEs
SM17-NGS-R1	CAACTATGGAGTGAAATCTCTTGTC	NGS primer for Base Editing with z-iSpyMacas9 BEs
SM17-NGS-F2	AGTCAAGCTCCAGCAATCACGACCTG	NGS primer for Base Editing with z-iSpyMacas9 BEs
SM17-NGS-R2	CACGATTGGAGTGAAATCTCTTGTC	NGS primer for Base Editing with z-iSpyMacas9 BEs
SM17-NGS-F3	AGTTCCGCTCCAGCAATCACGACCTG	NGS primer for Base Editing with z-iSpyMacas9 BEs
SM17-NGS-R3	CCAACATGGAGTGAAATCTCTTGTC	NGS primer for Base Editing with z-iSpyMacas9 BEs
SM17-NGS-F4	TTAGGCGCTCCAGCAATCACGACCTG	NGS primer for Base Editing with z-iSpyMacas9 BEs
SM17-NGS-R4	CACTCATGGAGTGAAATCTCTTGTC	NGS primer for Base Editing with z-iSpyMacas9 BEs
SM21-NGS-F1	TAGCTTGTGGAGAGTTGGGCACCCAC	NGS primer for Base Editing with z-iSpyMacas9 BEs
SM21-NGS-R1	CAAAAGGTGCTCAGTCTGTAGTGGAG	NGS primer for Base Editing with z-iSpyMacas9 BEs
SM21-NGS-F2	CTTGTAGTGGAGAGTTGGGCACCCAC	NGS primer for Base Editing with z-iSpyMacas9 BEs
SM21-NGS-R2	TATAATGTGCTCAGTCTGTAGTGGAG	NGS primer for Base Editing with z-iSpyMacas9 BEs
SM21-NGS-F3	CCGTCCGTGGAGAGTTGGGCACCCAC	NGS primer for Base Editing with z-iSpyMacas9 BEs
SM21-NGS-R3	TCATTCGTGCTCAGTCTGTAGTGGAG	NGS primer for Base Editing with z-iSpyMacas9 BEs
SM2-NGS-F1	ACAGTGGAATCGCCGCACGCAGATGAAG	For testing genome editing activity at SM2 site
SM2-NGS-R1	CAACTAGCTCCTCTAGGGACACCTCC	For testing genome editing activity at SM2 site
SM6/26-NGS-F1	AGTCATCCTGTAGGTCACATGGGTTG	For testing genome editing activity at SM6 and SM26 sites
SM6/26-NGS-R1	CAGGATCACAGTTGATTCCGCAAATTG	For testing genome editing activity at SM6 and SM26 sites
SM6/26-NGS-F2	AGTTCCCCTGTAGGTCACATGGGTTG	For testing genome editing activity at SM6 and SM26 sites
SM6/26-NGS-R2	CCAACACACAGTTGATTCCGCAAATTG	For testing genome editing activity at SM6 and SM26 sites
SM6/26-NGS-F3	TTAGGCCCTGTAGGTCACATGGGTTG	For testing genome editing activity at SM6 and SM26 sites
SM6/26-NGS-R3	CACTCACACAGTTGATTCCGCAAATTG	For testing genome editing activity at SM6 and SM26 sites
SM6/26-NGS-F4	TAGCTTCCTGTAGGTCACATGGGTTG	For testing genome editing activity at SM6 and SM26 sites
SM6/26-NGS-R4	CAAAAGCACAGTTGATTCCGCAAATTG	For testing genome editing activity at SM6 and SM26 sites
SM10-NGS-F1	CTTGTAGAATCGCCGCACGCAGATGAAG	For testing genome editing activity at SM10 site
SM10-NGS-R1	TATAATGCTCCTCTAGGGACACCTCC	For testing genome editing activity at SM10 site
SM10-NGS-F2	CCGTCCGAATCGCCGCACGCAGATGAAG	For testing genome editing activity at SM10 site
SM10-NGS-R2	TCATTCGCTCCTCTAGGGACACCTCC	For testing genome editing activity at SM10 site
SM10-NGS-F3	ACAGTGGAATCGCCGCACGCAGATGAAG	For testing genome editing activity at SM10 site
SM10-NGS-R3	CAACTAGCTCCTCTAGGGACACCTCC	For testing genome editing activity at SM10 site
SM10-NGS-F4	AGTCAAGAATCGCCGCACGCAGATGAAG	For testing genome editing activity at SM10 site
SM10-NGS-R4	CACGATGCTCCTCTAGGGACACCTCC	For testing genome editing activity at SM10 site
150-R221K-F	cttgagaaccttatcgctcagcttcc	For targeted mutagenesis to make pYPQ150-iSpymac
150-R221K-R	CTtacgagacttagaaagtctagcagaaag	For targeted mutagenesis to make pYPQ150-iSpymac
150-N394K-F	agagaggatcttcttagaaagcagagaa	For targeted mutagenesis to make pYPQ150-iSpymac
150-N394K-R	Cttcaacttaacaagaagctcctcggtt	For targeted mutagenesis to make pYPQ150-iSpymac
166-R221K-F	ctcgagaacctgatcgcccagc	For targeted mutagenesis to make pYPQ166-iSpymac
166-R221K-R	CTTccgagacttcgagagcctagcgg	For targeted mutagenesis to make pYPQ166-iSpymac
166-N394K-F	cgcgaggacctcctgaggaagc	For targeted mutagenesis to make pYPQ166-iSpymac
166-N394K-R	Cttgagcttcaccagcagctcctcc	For targeted mutagenesis to make pYPQ166-iSpymac

Supplemental Table 2. T-DNA expression constructs used in this study.

Name	Targeted rice gene	PAM (5'-->3')	gRNA	gRNA expression type	Purpose	Figure shown
pLR1812	OsPDS	TAAG	OsPDS-gRNA07	pOsU6-gRNA	Testing pco-SpyMacCas9 at targets associated with selected NAAN PAMs	Figure 2B
pLR1813	OsPDS	TAAC	OsPDS-gRNA08	pOsU3-gRNA	Testing pco-SpyMacCas9 at targets associated with selected NAAN PAMs	Figure 2B
pLR1814	OsPDS	GAAA	OsPDS-gRNA09	pOsU6-gRNA	Testing pco-SpyMacCas9 at targets associated with selected NAAN PAMs	Figure 2B
pLR1815	OsPDS	GAAG	OsPDS-gRNA11	pOsU6-gRNA	Testing pco-SpyMacCas9 at targets associated with selected NAAN PAMs	Figure 2B
pLR1817	OsPDS	TAAG	OsPDS-gRNA07	pOsU6-gRNA	Testing z-SpyMacCas9 at targets associated with selected NAAN PAMs	Figure 2B
pLR1818	OsPDS	TAAC	OsPDS-gRNA08	pOsU3-gRNA	Testing z-SpyMacCas9 at targets associated with selected NAAN PAMs	Figure 2B
pLR1819	OsPDS	GAAA	OsPDS-gRNA09	pOsU6-gRNA	Testing z-SpyMacCas9 at targets associated with selected NAAN PAMs	Figure 2B
pLR1820	OsPDS	GAAG	OsPDS-gRNA11	pOsU6-gRNA	Testing z-SpyMacCas9 at targets associated with selected NAAN PAMs	Figure 2B, 3A
pLR1822	OsPDS	TAAG	OsPDS-gRNA07	pOsU6-gRNA	Testing pco-iSpyMacCas9 at targets associated with selected NAAN PAMs	Figure 2B
pLR1823	OsPDS	TAAC	OsPDS-gRNA08	pOsU3-gRNA	Testing pco-iSpyMacCas9 at targets associated with selected NAAN PAMs	Figure 2B
pLR1824	OsPDS	GAAA	OsPDS-gRNA09	pOsU6-gRNA	Testing pco-iSpyMacCas9 at targets associated with selected NAAN PAMs	Figure 2B
pLR1825	OsPDS	GAAG	OsPDS-gRNA11	pOsU6-gRNA	Testing pco-iSpyMacCas9 at targets associated with selected NAAN PAMs	Figure 2B
pLR1827	OsPDS	TAAG	OsPDS-gRNA07	pOsU6-gRNA	Testing z-iSpyMacCas9 at targets associated with NAAN PAMs	Figure 2B, 2C
pLR1828	OsPDS	TAAC	OsPDS-gRNA08	pOsU3-gRNA	Testing z-iSpyMacCas9 at targets associated with NAAN PAMs	Figure 2B, 2C
pLR1829	OsPDS	GAAA	OsPDS-gRNA09	pOsU6-gRNA	Testing z-iSpyMacCas9 at targets associated with NAAN PAMs	Figure 2B, 2C, 3C
pLR1830	OsPDS	GAAG	OsPDS-gRNA11	pOsU6-gRNA	Testing z-iSpyMacCas9 at targets associated with NAAN PAMs	Figure 2B, 2C, 3B
pLR2218	OsPDS	AAAA	OsPDS-gRNA01	pOsU3-gRNA	Testing z-iSpyMacCas9 at targets associated with NAAN PAMs	Figure 2C
pLR2219	OsROC5	AAAA	OsROC5-gRNA01	pOsU6-gRNA	Testing z-iSpyMacCas9 at targets associated with NAAN PAMs	Figure 2C
pLR2220	OsPDS	AAAT	OsPDS-gRNA02	pOsU3-gRNA	Testing z-iSpyMacCas9 at targets associated with NAAN PAMs	Figure 2C
pLR2221	OsROC5	AAAT	OsROC5-gRNA02	pOsU3-gRNA	Testing z-iSpyMacCas9 at targets associated with NAAN PAMs	Figure 2C
pLR2222	OsPDS	AAAG	OsPDS-gRNA03	pOsU3-gRNA	Testing z-iSpyMacCas9 at targets associated with NAAN PAMs	Figure 2C
pLR2223	OsROC5	AAAG	OsROC5-gRNA03	pOsU6-gRNA	Testing z-iSpyMacCas9 at targets associated with NAAN PAMs	Figure 2C, 3D
pLR2224	OsPDS	AAAC	OsPDS-gRNA04	pOsU3-gRNA	Testing z-iSpyMacCas9 at targets associated with NAAN PAMs	Figure 2C
pLR2225	OsROC5	AAAC	OsROC5-gRNA04	pOsU6-gRNA	Testing z-iSpyMacCas9 at targets associated with NAAN PAMs	Figure 2C
pLR2226	OsPDS	TAAA	OsPDS-gRNA05	pOsU3-gRNA	Testing z-iSpyMacCas9 at targets associated with NAAN PAMs	Figure 2C
pLR2227	OsROC5	TAAA	OsROC5-gRNA05	pOsU6-gRNA	Testing z-iSpyMacCas9 at targets associated with NAAN PAMs	Figure 2C
pLR2228	OsPDS	TAAT	OsPDS-gRNA06	pOsU6-gRNA	Testing z-iSpyMacCas9 at targets associated with NAAN PAMs	Figure 2C
pLR2229	OsROC5	TAAT	OsROC5-gRNA06	pOsU3-gRNA	Testing z-iSpyMacCas9 at targets associated with NAAN PAMs	Figure 2C
pLR2230	OsROC5	TAAG	OsROC5-gRNA07	pOsU6-gRNA	Testing z-iSpyMacCas9 at targets associated with NAAN PAMs	Figure 2C
pLR2231	OsROC5	TAAC	OsROC5-gRNA08	pOsU3-gRNA	Testing z-iSpyMacCas9 at targets associated with NAAN PAMs	Figure 2C
pLR2232	OsROC5	GAAA	OsROC5-gRNA09	pOsU3-gRNA	Testing z-iSpyMacCas9 at targets associated with NAAN PAMs	Figure 2C
pLR2233	OsPDS	GAAT	OsPDS-gRNA10	pOsU6-gRNA	Testing z-iSpyMacCas9 at targets associated with NAAN PAMs	Figure 2C
pLR2234	OsROC5	GAAT	OsROC5-gRNA10	pOsU3-gRNA	Testing z-iSpyMacCas9 at targets associated with NAAN PAMs	Figure 2C
pLR2235	OsROC5	GAAG	OsROC5-gRNA11	pOsU6-gRNA	Testing z-iSpyMacCas9 at targets associated with NAAN PAMs	Figure 2C
pLR2236	OsPDS	GAAC	OsPDS-gRNA12	pOsU6-gRNA	Testing z-iSpyMacCas9 at targets associated with NAAN PAMs	Figure 2C
pLR2237	OsROC5	GAAC	OsROC5-gRNA12	pOsU3-gRNA	Testing z-iSpyMacCas9 at targets associated with NAAN PAMs	Figure 2C
pLR2238	OsPDS	CAAA	OsPDS-gRNA13	pOsU3-gRNA	Testing z-iSpyMacCas9 at targets associated with NAAN PAMs	Figure 2C
pLR2239	OsROC5	CAAA	OsROC5-gRNA13	pOsU6-gRNA	Testing z-iSpyMacCas9 at targets associated with NAAN PAMs	Figure 2C
pLR2240	OsPDS	CAAT	OsPDS-gRNA14	pOsU3-gRNA	Testing z-iSpyMacCas9 at targets associated with NAAN PAMs	Figure 2C
pLR2241	OsROC5	CAAT	OsROC5-gRNA14	pOsU3-gRNA	Testing z-iSpyMacCas9 at targets associated with NAAN PAMs	Figure 2C
pLR2242	OsPDS	CAAG	OsPDS-gRNA15	pOsU3-gRNA	Testing z-iSpyMacCas9 at targets associated with NAAN PAMs	Figure 2C
pLR2243	OsROC5	CAAG	OsROC5-gRNA15	pOsU6-gRNA	Testing z-iSpyMacCas9 at targets associated with NAAN PAMs	Figure 2C
pLR2244	OsPDS	CAAC	OsPDS-gRNA16	pOsU3-gRNA	Testing z-iSpyMacCas9 at targets associated with NAAN PAMs	Figure 2C
pLR2245	OsROC5	CAAC	OsROC5-gRNA16	pOsU3-gRNA	Testing z-iSpyMacCas9 at targets associated with NAAN PAMs	Figure 2C
pLR2294	OsPDS	GAAA	OsPDS-gRNA09	pOsU6-gRNA	Testing C to T base editing with hAID-z-iSpyMacCas9-D10A-UGI base editor	Figure 4B, 4E
pLR2295	OsPDS	GAAA	OsPDS-gRNA09	pOsU6-gRNA	Testing C to T base editing with z-iSpyMacCas9-D10A-PmCDA-UGI base editor	Figure 4B, 4E
pLR2300	OsPDS	GAAG	OsPDS-gRNA11	pOsU6-gRNA	Testing A to G base editing with wtTadA-TadA*-z-iSpyMacCas9-D10A base editor	Figure 5B
pLR2764	OsROC5	AAAG	OsROC5-gRNA03	pOsU6-gRNA	Testing C to T base editing with hAID-z-iSpyMacCas9-D10A-UGI base editor	Figure 4B, 4C
pLR2765	OsROC5	AAAG	OsROC5-gRNA03	pOsU6-gRNA	Testing C to T base editing with z-iSpyMacCas9-D10A-PmCDA-UGI base editor	Figure 4B, 4C
pLR2767	OsROC5	AAAG	OsROC5-gRNA03	pOsU6-gRNA	Testing A to G base editing with wtTadA-TadA*-z-iSpyMacCas9-D10A base editor	Figure 5B
pLR2768	OsROC5	TAAA	OsROC5-gRNA05	pOsU6-gRNA	Testing C to T base editing with hAID-z-iSpyMacCas9-D10A-UGI base editor	Figure 4B, 4D
pLR2769	OsROC5	TAAA	OsROC5-gRNA05	pOsU6-gRNA	Testing C to T base editing with z-iSpyMacCas9-D10A-PmCDA-UGI base editor	Figure 4B, 4D, 4G, Supp. Fig. 1
pLR2771	OsROC5	TAAA	OsROC5-gRNA05	pOsU6-gRNA	Testing A to G base editing with wtTadA-TadA*-z-iSpyMacCas9-D10A base editor	Figure 5B, 5C
pLR2772	OsROC5	CAAA	OsROC5-gRNA13	pOsU6-gRNA	Testing C to T base editing with hAID-z-iSpyMacCas9-D10A-UGI base editor	Figure 4B, 4F
pLR2773	OsROC5	CAAA	OsROC5-gRNA13	pOsU6-gRNA	Testing C to T base editing with z-iSpyMacCas9-D10A-PmCDA-UGI base editor	Figure 4B, 4F, 4H, Supp. Fig. 2
pLR2775	OsROC5	CAAA	OsROC5-gRNA13	pOsU6-gRNA	Testing A to G base editing with wtTadA-TadA*-z-iSpyMacCas9-D10A base editor	Figure 5B

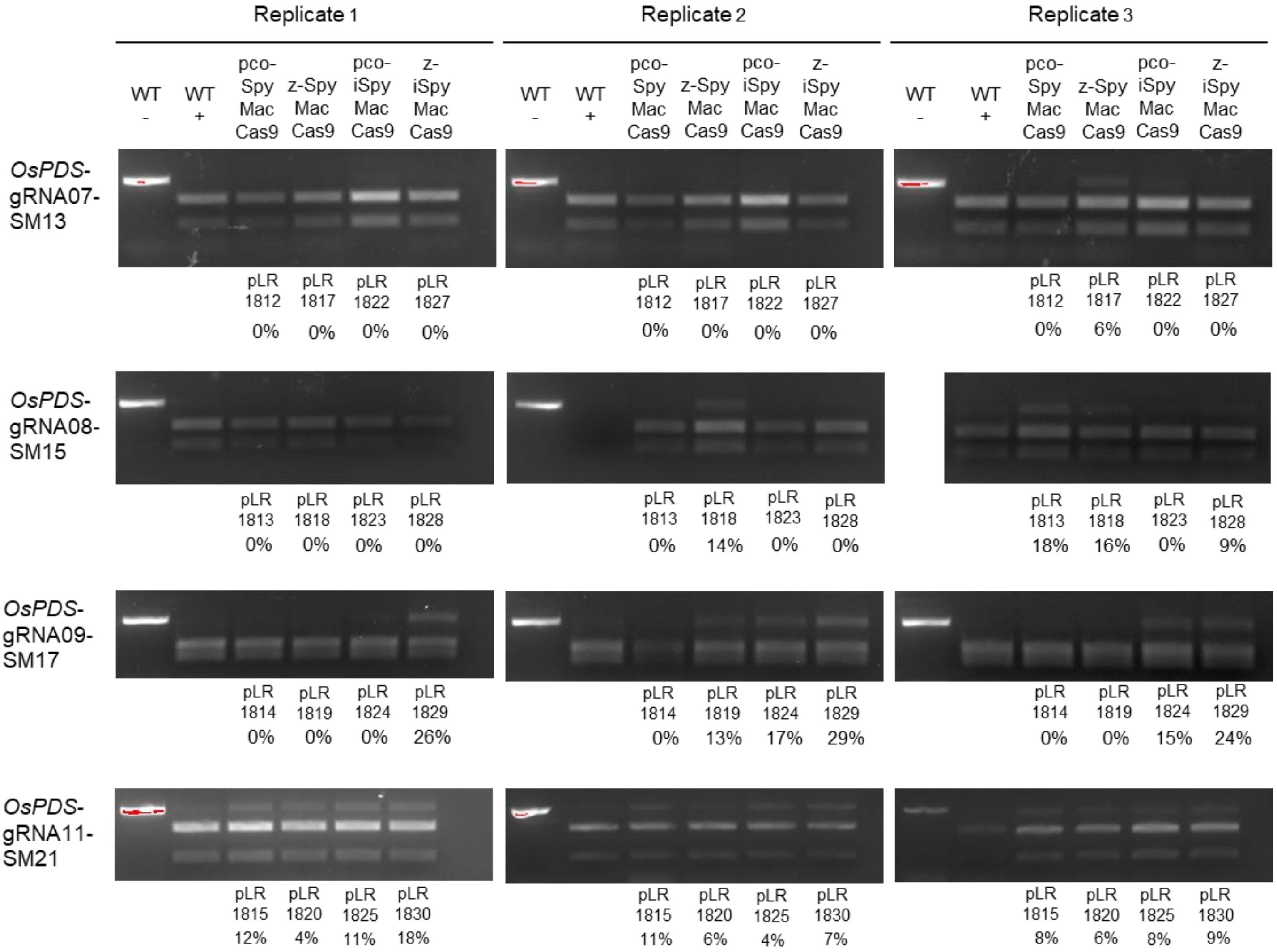

Supplemental Figure 1. Raw RFLP data of testing 4 (i)SpyMacCas9 variants at 4 target sites in 3 replicates.

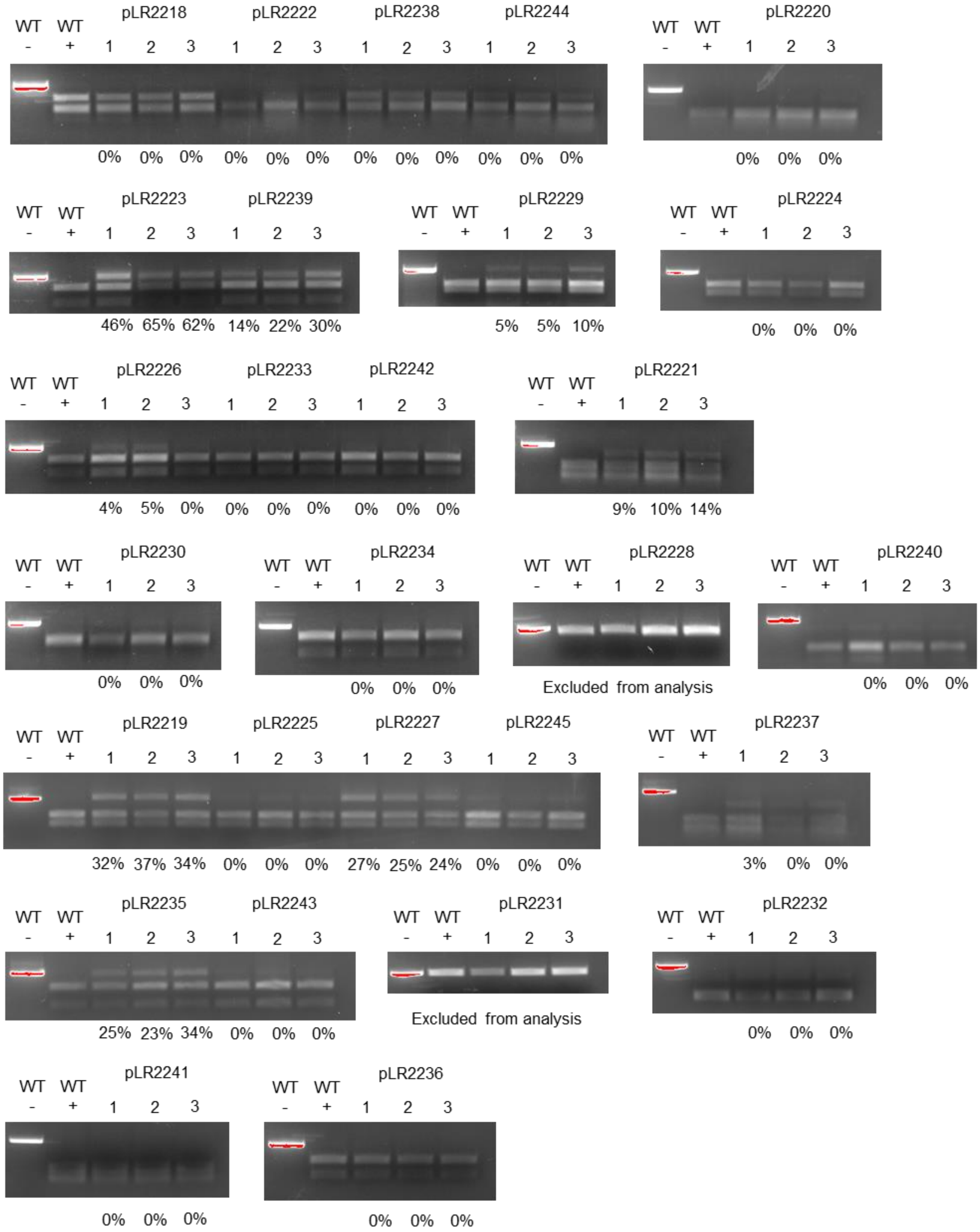

Supplemental Figure 2: Raw RFLP data of testing z-iSpyMacCas9 at additional 28 target sites in 3 replicates. In total, the nuclease activity of z-iSpyMacCas9 was screened 32 target sites. Data from two target sites were excluded from analysis due to enzyme inactivity.

```
pLR2769:     z-iSpyMacCas9-PmCDA1-UGI + OsROC5-sgRNA05-SM10
             AGCGCGTTCTCGTGCCGCTCCAGTTGCGTCTTGGTAAAACGAAGATCGCGGC   WT(OsPDS)
pLR2769-7
Allele 1:    AGCGCGTT-----------------------TGGTAAAACGAAGATCGCGGC   -23bp
Allele 2:    AGCGCGTTCT(64-bp del)CGTGTCAGGAAATTGCACGAAACGAA        -64bp
pLR2769-13
Allele 1:    AGCGCGTTTCTCGTGCCGCTCCAGTTGCGTCTTGGTAAAACGAAGATCGCGGC  +1bp
Allele 2:    AGCGCGTTCCTCGTGCCGCTCCAGTTGCGTCTTGGTAAAACGAAGATCGCGGC  +1bp
```

Supplemental Figure 3. T0 indel mutants generated by PmCDA1 based iSpyMacCas9 CBE with OsROC5-sgRNA05. The PAM is highlighted in red and the protospacer is highlighted in blue.

pLR2773:	z-iSpyMacCas9-PmCDA1-UGI + *OsROC5*-sgRNA13-SM26	
	TGGCGCTGCAGCGTCGCAAGCCAACGCCTGGCGCCAAAGGCGAGACCAGA	WT(OsPDS)
pLR2773-2		
Allele 1:	TGGCGCTGCAGCGTCG-AAGCCAACGCCTGGCGCCAAAGGCGAGACCAGA	-1bp
Allele 2:	TGGCGCTGCAGCGTCGTAAGCCAACGCCTGGCGCCAAAGGCGAGACCAGA	C2T
pLR2773-8		
Allele 1:	TGGCGCTGCAGCGTCGCAAGCCAACGCCTGGCGCCAAAGGCGAGACCAGA	WT
Allele 2:	TGGCGCTGCAGCGAAGTCAACC----CCTGGCGCCAAAGGCGAGACCAGA	T-2A; C-2A; C2T; A3C; G5A; -4bp
pLR2773-20		
Allele 1:	TGGCGCTGCAGCGTCGCAAGCCAACGCCTGGCGCCAAAGGCGAGACCAGA	WT
Allele 2:	TGGCGCTGCAGCGTCGGAAGCCAAC-----GCGCCAAAGGCGAGACCAGA	C2G; -5bp
pLR2773-22		
Allele 1:	TGGCGCTGCAG---------------------------------ACCAGA	-33bp
Allele 2:	TGGCGCTGCAGCGTCGGAAGCCAACGCCTGGCGCCAAAGGCGAGACCAGA	C2G
pLR2773-25		
Allele 1:	TGGCGCTGCAGCGTCGCAAGC---------CAACGCCTGGCGCCAAAGGC	WT
Allele 2:	TGGCGCTGCAGCGTCGCAAGCGTCCTAAGCCAACGCCTGGCGCCAAAGGC	+9bp

Supplemental Figure 4. Additional mutated T0 lines induced by PmCDA1 based iSpyMacCas9 CBE with OsROC5-sgRNA13. The PAM is highlighted in red and the protospacer is highlighted in blue.
