# Supplementary material for: Expanding plant genome-editing scope by an engineered iSpyMacCas9 system that targets A-rich PAM sequences
Source: Plant Commun. 2020 Jul 22;2(2):100101. doi: 10.1016/j.xplc.2020.100101 (PMC8060698; doi:10.1016/j.xplc.2020.100101)
Supplement: Document S2. Article plus Supplemental Information [file mmc2.pdf]

Please cite this article in press as: Sretenovic et al., Expanding Plant Genome-Editing Scope by an Engineered iSpyMacCas9 System That Targets A-Rich PAM Sequences, Plant Communications (2020), https://doi.org/10.1016/j.xplc.2020.100101

Plant Communications
Resource Article

CellPress Partner Journal

Expanding Plant Genome-Editing Scope by an Engineered iSpyMacCas9 System That Targets A-Rich PAM Sequences

Simon Sretenovic[1,7], Desuo Yin[1,2,7], Adam Levav[1,3], Jeremy D. Selengut[4], Stephen M. Mount[5] and Yiping Qi[1,6,*]

[1]Department of Plant Science and Landscape Architecture, University of Maryland, College Park, MD 20742, USA
[2]Hubei Academy of Agricultural Sciences, Wuhan 430064, China
[3]Montgomery Blair High School, Silver Spring, MD 20901, USA
[4]Center for Bioinformatics and Computational Biology, University of Maryland, College Park, MD 20742, USA
[5]Department of Cell Biology and Molecular Genetics, University of Maryland, College Park, MD 20742, USA
[6]Institute for Bioscience and Biotechnology Research, University of Maryland, Rockville, MD 20850, USA
[7]These authors contributed equally to this work.
***Correspondence: Yiping Qi (yiping@umd.edu)**
https://doi.org/10.1016/j.xplc.2020.100101

ABSTRACT

The most popular CRISPR-SpCas9 system recognizes canonical NGG protospacer adjacent motifs (PAMs). Previously engineered SpCas9 variants, such as Cas9-NG, favor G-rich PAMs in genome editing. In this manuscript, we describe a new plant genome-editing system based on a hybrid iSpyMacCas9 platform that allows for targeted mutagenesis, C to T base editing, and A to G base editing at A-rich PAMs. This study fills a major technology gap in the CRISPR-Cas9 system for editing NAAR PAMs in plants, which greatly expands the targeting scope of CRISPR-Cas9. Finally, our vector systems are fully compatible with Gateway cloning and will work with all existing single-guide RNA expression systems, facilitating easy adoption of the systems by others. We anticipate that more tools, such as prime editing, homology-directed repair, CRISPR interference, and CRISPR activation, will be further developed based on our promising iSpyMac-Cas9 platform.

Key words: plant genome editing, iSpyMacCas9, PAM, cytosine base editing, adenine base editing

Sretenovic S., Yin D., Levav A., Selengut J.D., Mount S.M., and Qi Y. (2020). Expanding Plant Genome-Editing Scope by an Engineered iSpyMacCas9 System That Targets A-Rich PAM Sequences. Plant Comm. **1**, 100101.

INTRODUCTION

The rapid development of CRISPR-Cas9, -Cas12a, and -Cas12b systems has greatly accelerated genome-editing applications in plants (Zhang et al., 2019). The most popular *Streptococcus pyogenes* Cas9 (SpCas9 or SpyCas9) system recognizes simple NGG protospacer adjacent motifs (PAMs) (N = A, T, C, G) (Jinek et al., 2012). Despite its widespread use, the SpCas9 system has a limited targeting scope due to its NGG PAM requirement. Over the past few years, efforts to expand the scope of genome editing in plants have mainly focused on the assessment of different Cas orthologs and variants that possess altered PAM requirements other than NGG. For example, the Cas9 orthologs *Streptococcus thermophilus* Cas9 (StCas9) (Garneau et al., 2010; Muller et al., 2016) and *Staphylococcus aureus* Cas9 (SaCas9) (Ran et al., 2015) were shown to recognize PAMs of NNAGAAW (W = A, T) and NNGRRN (R = A or G), respectively, in *Arabidopsis* (Steinert et al., 2015), although the study did not fully validate the PAM scope of StCas9 and SaCas9. SaCas9 was demonstrated in additional plant species such as *Nicotiana benthamiana* (Kaya et al., 2017), rice (Kaya et al., 2016), and citrus (Jia et al., 2017). The engineered SpCas9 variant, SpCas9-VQR (Kleinstiver et al., 2015), was confirmed to edit PAMs of NGAN or NGNG in rice (Hu et al., 2016, 2018b). SpCas9-NG (Nishimasu et al., 2018) was shown to edit NG PAMs in rice and *Arabidopsis* (Endo et al., 2019; Ge et al., 2019; Hua et al., 2019; Ren et al., 2019; Zhong et al., 2019). In addition, type IV CRISPR systems, such as Cas12a and Cas12b, offer recognition of T-rich PAMs (Zhang et al., 2019), and the LbCas12a-RR variant enables the recognition of C-rich PAMs (Zhong et al., 2018) in plants.

Published by the Plant Communications Shanghai Editorial Office in association with Cell Press, an imprint of Elsevier Inc., on behalf of CSPB and IPPE, CAS.

This is an open access article under the CC BY license (http://creativecommons.org/licenses/by/4.0/).

Please cite this article in press as: Sretenovic et al., Expanding Plant Genome-Editing Scope by an Engineered iSpyMacCas9 System That Targets A-Rich PAM Sequences, Plant Communications (2020), https://doi.org/10.1016/j.xplc.2020.100101

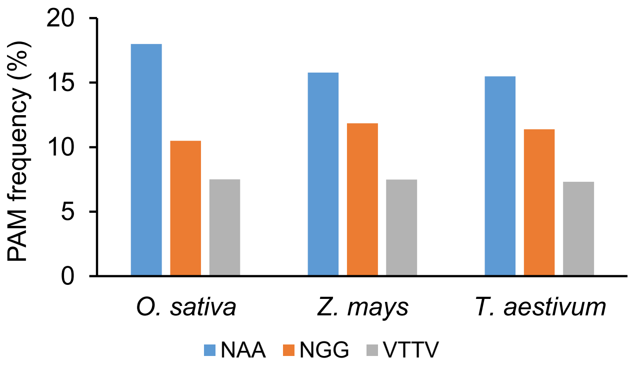

Figure 1. Genome-wide Analysis of PAM Frequencies in Rice, Maize, and Wheat.
In silico analysis of NAA, NGG, and VTTV (V = A, C, G) PAMs in the genomes of rice (*O. sativa*), maize (*Z. mays*), and wheat (*T. aestivum*).

However, a CRISPR-Cas9 system for plant genome engineering capable of recognizing A-rich PAMs remains elusive.

CRISPR-Cas systems are most effective in introducing indel (insertion and deletion) mutations through non-homologous end joining (Puchta, 2005). However, precise crop breeding often relies on the ability to swap alleles at single-base accuracy, which can be achieved by base editors. Currently, cytosine base editor (CBE) and adenine base editor (ABE) systems, first developed in human cells, have been applied in plants (Eid et al., 2018; Molla and Yang, 2019; Gurel et al., 2020). Among the multiple CBE configurations, the most efficient and popular one is base editor 3 (BE3) (Komor et al., 2016). In recent years, researchers have reported successful application of this system in rice (Li et al., 2017a; Lu and Zhu, 2017; Ren et al., 2017), wheat, and maize (Zong et al., 2017). Besides the APOBEC1 cytidine deaminase used in BE3, other deaminases have also been reported in plants, including *Petromyzon marinus* cytidine deaminase (PmCDA1) (Shimatani et al., 2017), a variant of human activation-induced cytidine deaminase (hAID*Δ) (Ren et al., 2018) and human APOBEC3A (Zong et al., 2018). We recently showed that the PmCDA1 base editor is much more efficient than the APOBEC1 base editor, regardless of whether it is expressed as a single transcript unit (Tang et al., 2018) or coupled with Cas9-NG for targeting relaxed NG PAM sites (Zhong et al., 2019). Besides introducing missense and nonsense mutations, CBE systems were also demonstrated for altering splicing sites in plants (Xue et al., 2018; Li et al., 2019b).

Development of the first ABE system was based on an impressive study that applied seven rounds of extensive protein evolution to the bacterial tRNA adenosine deaminase TadA to generate a highly efficient DNA base editor, ABE-7.10, for A to G base changes (Gaudelli et al., 2017). More recently, an engineered ABE system, ABEmax, was reported to have enhanced base editing activity in human cells (Koblan et al., 2018). The application of ABE systems in plants has been reported in *Arabidopsis*, *Brassica napus* (Kang et al., 2018), rice (Yan et al., 2018; Negishi et al., 2019; Molla et al., 2020), and wheat (Li et al., 2018). The ABE systems are also potentially useful for introducing mutations at A-T rich *cis*-regulatory elements for regulating gene expression. Both CBE and ABE systems fill a major technology gap in precise plant genome editing. However, the base editing scope is restricted by the PAM requirements of Cas9 proteins in use.

To expand the scope of targeted mutagenesis and base editing, Chatterjee et al. (2020) swapped the PAM interacting (PI) domain of SpyCas9 with the PI domain from *Streptococcus macacae* Cas9 (SmacCas9), which recognizes NAA PAMs. Interestingly, the hybrid SpyMacCas9 gained NAA PAM-targeting capability while retaining the nuclease activity of SpyCas9 *in vitro* and in human cells. In addition, an improved SpyMacCas9 platform displaying high nuclease activity, named iSpyMacCas9, was further engineered (Chatterjee et al., 2020). Hence, we were very intrigued by the great potential of the (i)SpyMacCas9 system in broadening the genome-editing scope of CRISPR-Cas9 in plants. Prior to the publication of the human cell study (Chatterjee et al., 2020), we decided to develop (i)SpyMacCas9 systems for targeted mutagenesis, C to T base editing, and A to G base editing in plants.

RESULTS

Genome-wide PAM Analysis in Rice, Maize, and Wheat

To assess the potential targeting scope of SpyMacCas9 and iSpyMacCas9 systems in major crops, we conducted an *in silico* PAM analysis of the genomes of rice (*Oryza sativa*), maize (*Zea mays*), and wheat (*Triticum aestivum*). We compared the frequencies of NAA PAMs with those of NGG PAMs, which are editable by SpyCas9, or VTTV PAMs, which are largely editable by FnCas12a (Zong et al., 2018). The results showed that in these three major crops, NAA PAMs occur far more frequently than NGG PAMs or VTTV PAMs (Figure 1). Furthermore, (i) SpyMacCas9 could in principle target twice as many sites in the genomes of rice, maize, and wheat than FnCas12a, although both favor A-T-rich PAMs (Figure 1). Hence, there is a great potential to develop (i)SpyMacCas9 genome-editing systems in plants due to their promise in greatly expanding the targeting scope.

Assessment of iSpyMacCas9 Systems in Rice Cells

We synthesized a rice codon-optimized PI domain from SmacCas9 and replaced the corresponding PI domain in two versions of SpyCas9, pcoCas9 (Li et al., 2013), and zCas9 (Xing et al., 2014), which generated pco-SpyMacCas9 and z-SpyMacCas9. R221K and N394K mutations, previously identified through deep mutational scans, can increase SpyCas9 nuclease activity (Spencer and Zhang, 2017). We further introduced these two mutations to create pco-iSpyMacCas9 and z-iSpyMacCas9. Hybrid SpyMacCas9 and iSpyMacCas9 protein domains are presented in Figure 2A. Four independent target sites with NAA PAMs in rice were chosen for the initial assessment of these four hybrid Cas9 systems in rice protoplasts. Three-way Gateway recombination was used to generate the expression vector with the SpyMacCas9 entry vector (Figure 2A), the single-guide RNA (sgRNA) entry vector pYPQ141D containing an OsU3 promoter for sgRNA expression, and the destination vector pYPQ203 containing a ZmUbi1 promoter for Cas9 expression (Lowder et al., 2015). The resulting T-DNA vectors were used for rice protoplast transformation. The protoplast results showed that two out of four NAA PAM sites were edited,

Please cite this article in press as: Sretenovic et al., Expanding Plant Genome-Editing Scope by an Engineered iSpyMacCas9 System That Targets A-Rich PAM Sequences, Plant Communications (2020), https://doi.org/10.1016/j.xplc.2020.100101

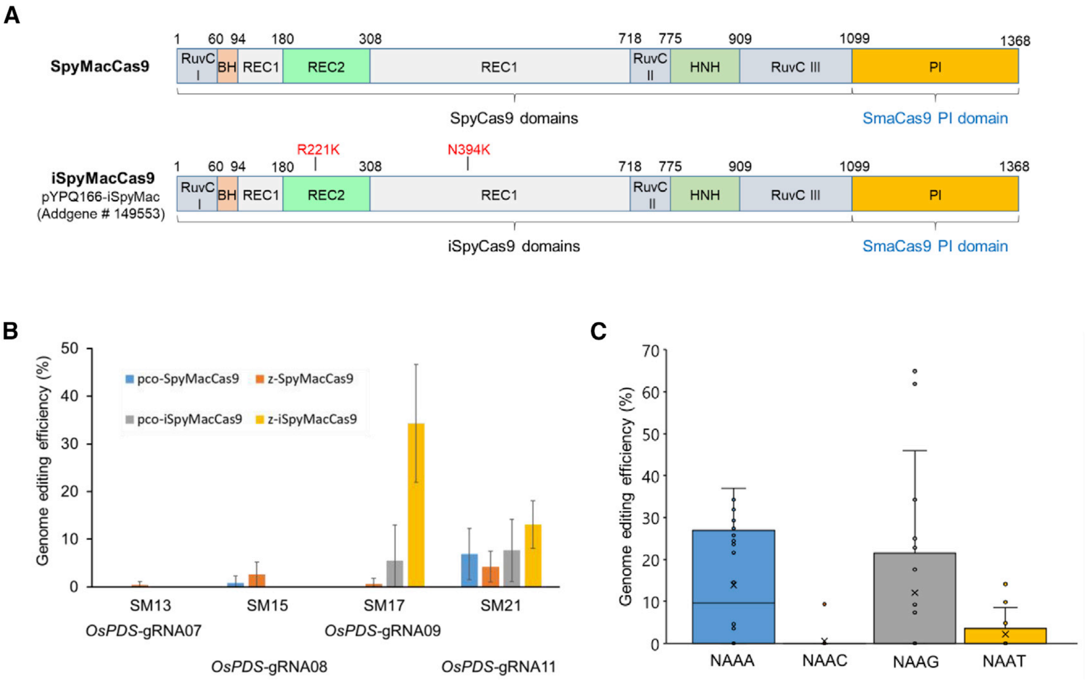

Figure 2. Development of an iSpyMacCas9 Plant Genome-Editing Toolbox and Its Assessment in Rice.

(A) Protein domains of the hybrid SpyMacCas9 and iSpyMacCas9 nucleases, which were generated based on two codon-optimized SpyCas9 versions, pcoCas9 and zCas9. The z-iSpyMacCas9 entry clone was deposited into Addgene (no. 149553).

(B) Genome-editing efficiency of four SpyMacCas9 systems at four target sites in rice protoplasts. Error bars represent the standard deviation of three independent experiments.

(C) Genome-editing efficiency of z-iSpyMacCas9 at 32 target sites with NAAA, NAAT, NAAG, and NAAC PAMs in rice protoplasts.

and z-iSpyMacCas9 appeared to be most potent, resulting in an editing frequency of >30% at one target site (Figure 2B and Supplemental Figure 1). Thus, we chose z-iSpyMacCas9 as our default iSpyMacCas9 system for all remaining experiments.

Our data suggested that iSpyMacCas9 may recognize PAMs that are more complex than NAA in rice. Therefore, we sought to investigate iSpyMacCas9 PAM requirements in a more comprehensive way. To accomplish this, we targeted a total of 32 sites in the rice genome associated with all 16 possible combinations of NAAN PAMs in duplicates. The protospacers were between 19 and 21 bp long, had a median GC% of 50%, and 90% of all the protospacers had a GC content between 30% and 70%. We assessed these constructs in rice protoplasts. The data indicated that iSpyMacCas9 could edit most NAAA and NAAG PAM sites but displayed low editing efficiencies at NAAC and NAAT PAM sites (Figure 2C and Supplemental Figure 2). Our data, along with recent reports in human cells (Chatterjee et al., 2020) and rabbit cells (Liu et al., 2019), suggest that while iSpyMacCas9 may generally target NAAN PAMs, its activity is greatly affected by the fourth nucleotide within the PAMs. Based on the editing data, we concluded that iSpyMacCas9 prefers NAAR (R = A, G) PAMs in rice.

Generation of Stably Edited T0 Rice Lines with iSpyMacCas9

We next evaluated (i)SpyMacCas9's ability to generate *OsPDS*- and *OsROC5*-targeted mutants in stable transgenic rice lines using *Agrobacterium*-mediated T-DNA transformation. *OsPDS* encodes phytoene desaturase, an important enzyme in the carotenoid biosynthesis pathway (Miki and Shimamoto, 2004). *OsROC5,* rice outermost cell-specific gene 5, is responsible for leaf rolling control (Zou et al., 2011). We first targeted a site with OsPDS-sgRNA11 for an NAAG PAM. We compared SpyMacCas9 and iSpyMacCas9, which resulted in one out of five (20%) and four out of seven (57.1%) edited T0 lines, respectively (Table 1, Figure 3A and 3B). These data confirmed our earlier observation in rice protoplasts that iSpyMacCas9 showed higher editing efficiency than SpyMacCas9 (Figure 2B). We then focused on iSpyMacCas9 and evaluated two additional T-DNA constructs with sgRNAs targeting NAAA and NAAG PAMs in *OsPDS* and *OsROC5*, respectively. At the OsPDS-sgRNA09 site, 6 out of 16 (37.5%) T0 lines were edited (Table 1 and Figure 3C). At the OsROC5-sgRNA03 site, eight out of nine (88.9%) T0 lines were edited (Table 1 and Figure 3D), suggesting very high editing efficiency. In both cases, T0 plants with biallelic edits were readily identified (Figure 3C and 3D). Taken collectively, these data demonstrated that z-iSpyMacCas9 is capable of generating rice mutants at NAAR PAM sites with high efficiency.

Development of Two C to T Base Editing Systems with iSpyMacCas9

We reasoned that iSpyMacCas9, with its A-rich PAM requirements, would have great potential in expanding the base editing scope in plants. To develop efficient CBEs based on iSpyMacCas9 in plants, we compared a hyperactive hAID mutant and PmCDA1, two potent cytidine deaminases (Nishida et al., 2016;

Please cite this article in press as: Sretenovic et al., Expanding Plant Genome-Editing Scope by an Engineered iSpyMacCas9 System That Targets A-Rich PAM Sequences, Plant Communications (2020), https://doi.org/10.1016/j.xplc.2020.100101

Constructs	PAM Index	Targeted Rice Sites	Cas9 Variants	Tested TO Lines	Mutated TO Lines (Number; Ratio)	Biallelic Mutation Lines (Number; Ratio)
pLR1820	GAAG	OsPDS-sgRNA11-SM21	z-SpyMacCas9	5	1; 20%	0; 0.0%
pLR1829	GAAA	OsPDS-sgRNA09-SM17	z-iSpyMacCas9	16	6; 37.5%	1; 6.3%
pLR1830	GAAG	OsPDS-sgRNA11-SM21	z-iSpyMacCas9	7	4; 57.1%	1; 14.2%
pLR2223	AAAG	OsROC5-sgRNA3-SM6	z-iSpyMacCas9	9	8; 88.9%	8; 88.9%

Table 1. Summary of Targeted Mutagenesis by (i)SpyMacCas9 in Rice T0 Lines.

Ren et al., 2018). The efficient BE3 configuration was chosen, and an iSpyMacCas9-D10A nickase and a uracil DNA glycosylase inhibitor were used (Komor et al., 2016) (Figure 4A). These two iSpyMacCas9 CBE systems were configured into Gateway-compatible vectors and used for generating T-DNA expression vectors by Gateway recombination. They were first tested at four target sites in rice protoplasts, and C to T base changes were assessed with next-generation sequencing (NGS). PmCDA1 resulted in a much higher C to T conversion frequency than hAID at the OsROC5-sgRNA03 site (Figure 4B). At the three additional sites, comparable editing frequencies were observed for both iSpyMacCas9 CBE systems (Figure 4B). Further analysis of these four target sites revealed a deamination window preference of the two iSpyMacCas9 CBE systems. The PmCDA1-based CBE preferably edited cytidines located at the 5′ end of the target sites, while the hAID-based CBE demonstrated a broader targeting window (5–12 nt from the 5′ end) with the highest activity in the middle of the same target sites (Figure 4C–4F). We tested the PmCDA1-based iSpyMacCas9 CBE system in stable rice plants. At the OsROC5-sgRNA05 site, five out 16 T0 lines had precise C to T base changes (Table 2), with lines no. 09 and 10 carrying the C3T monoallelic base conversion, and line no. 04 carrying the C2T monoallelic base conversion and C3T;C5T biallelic base conversions (Figure 4G). Some T0 lines were identified with indel mutations, resulting from non-homologous end joining DNA repair (Supplemental Figure 3). At the OsROC5-sgRNA13 site, six out of 18 T0 lines showed a C to G base transversion change (Table 2 and Figure 4H). Within this T0 population, four lines showed C to T or C to G base editing along with indel mutations (Supplemental Figure 4). Hence, we demonstrated two iSpyMacCas9-based CBE systems in rice.

A to G Base Editing with an iSpyMacCas9 ABE

To enable A to G base editing at NAAR PAM sites, we developed an iSpyMacCas9 ABE vector based on the adenosine deaminase configuration from the highly active ABEmax system (Koblan et al., 2018) (Figure 5A). This iSpyMacCas9 ABE system was tested at four target sites in rice protoplasts, and A to G base conversions were detected by NGS at all target sites (Figure 5B). To assess whether this iSpyMacCas9 ABE system can efficiently generate A to G base changes in stable rice lines, we focused on the construct with OsROC5-sgRNA5 since it showed an average base editing efficiency among all four constructs in the protoplast assay (Figure 5B). We then generated and genotyped a total of 24 T0 lines with this ABE construct. Among these T0 lines, three base-edited lines were identified (Table 2), all carrying a monoallelic A9G base change (Figure 5C), indicating an A to G editing efficiency of 12.5%. Notably, no indel mutations were identified in T0 lines (Table 2). Thus, we established an efficient iSpyMacCas9-based ABE system for high purity A to G base editing at NAAR PAM sites in rice.

DISCUSSION

Expanding the targeting scope of CRISPR-Cas9 has been pursued heavily in the field of genome editing so that many more sequences in a genome will become editable. There are two major routes for achieving Cas9 with altered PAM requirements, one relying on the investigation of Cas9 orthologs that have naturally evolved different PAM preferences than SpCas9 and the other relying on protein engineering. Within the protein engineering route, there are three approaches. The first approach is based on protein evolution. For example, xCas9 was generated with phage-assisted continuous evolution to target relaxed NG PAMs (Hu et al., 2018a). The second approach is based on structure-directed mutagenesis. For example, Cas9-NG, also claimed to target NG PAMs, was engineered in this manner (Nishimasu et al., 2018). The third approach is based on domain swap between Cas9 orthologs. This is how the SpyMacCas9 system was established (Chatterjee et al., 2020). For these engineered SpCas9 variants, all the initial assessments were made in human cells. Interestingly, plant researchers have recently tested xCas9 and found that it has extremely low editing efficiency at NG PAMs in plants (Wang et al., 2018; Li et al., 2019a; Hua et al., 2019; Zhong et al., 2019; Zeng et al., 2020). While Cas9-NG can edit many NG PAM sites in plants (Endo et al., 2019; Hua et al., 2019; Ren et al., 2019; Zhong et al., 2019; Zeng et al., 2020), it prefers NGT PAMs rather than NGV PAMs, and its activity is very low at NGV PAMs (Zhong et al., 2019). Furthermore, its activity at canonical NGG PAM sites is much lower than that of the wild-type (WT) SpCas9 (Zhong et al., 2019). Thus, it is necessary to systematically evaluate the PAM requirements for a novel Cas9 protein in plants before its wide adoption by the plant research community.

In this study, we assessed the PAM requirements of the iSpyMacCas9 system in rice and found that it recognized NAAR PAMs. Our observation is in general agreement with the original report of iSpyMacCas9 with NAA PAMs *in vitro* and in human cells

Please cite this article in press as: Sretenovic et al., Expanding Plant Genome-Editing Scope by an Engineered iSpyMacCas9 System That Targets A-Rich PAM Sequences, Plant Communications (2020), https://doi.org/10.1016/j.xplc.2020.100101

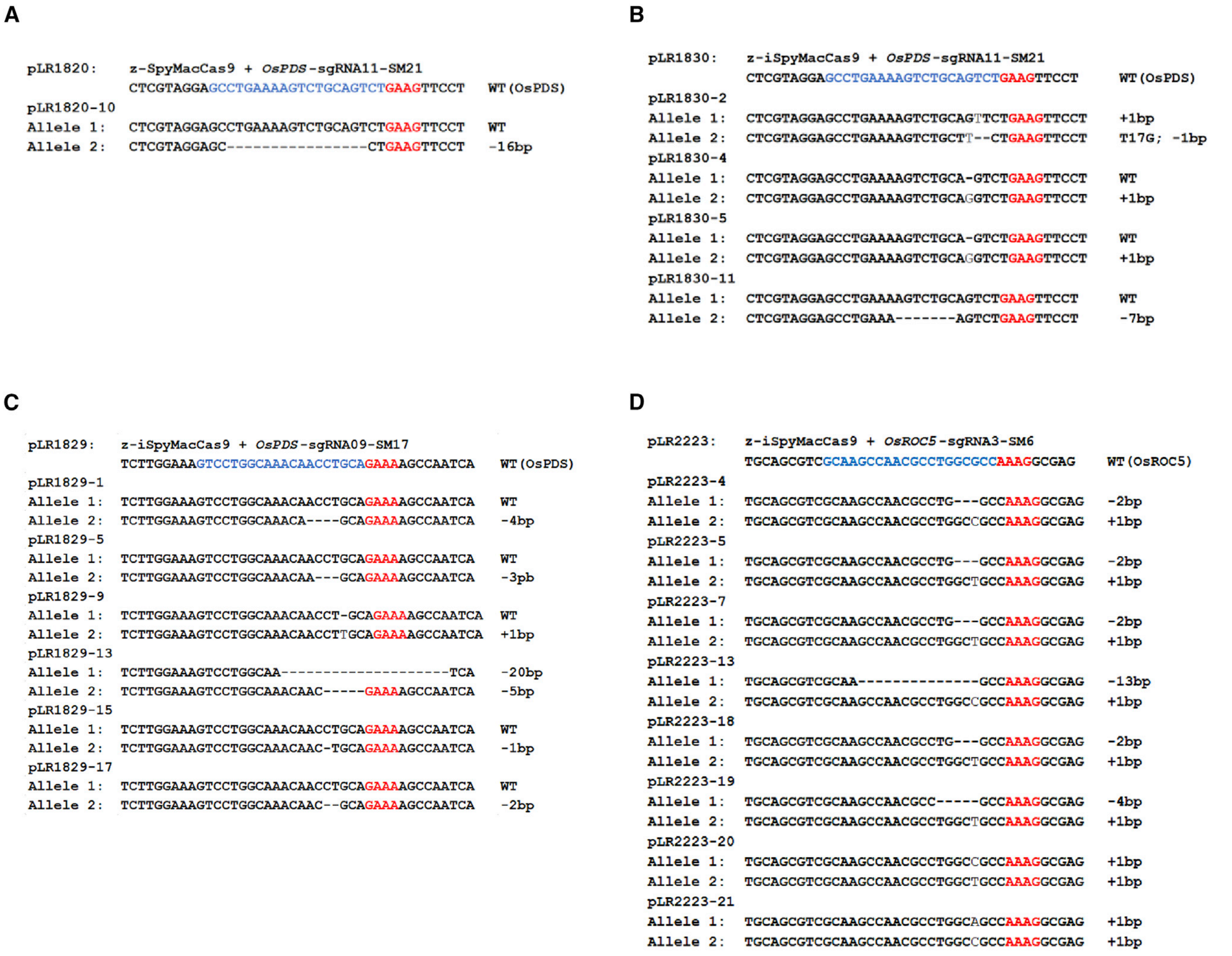

Figure 3. Assessment of (i)SpyMacCas9 for Targeted Mutagenesis in Rice T0 Lines.

(A) Genotype of a targeted T0 mutant by SpyMacCas9 at the OsPDS-sgRNA11 (SM21) site.

(B) Genotypes of targeted T0 mutants by iSpyMacCas9 at the OsPDS-sgRNA11 (SM21) site.

(C) Genotypes of targeted T0 mutants by iSpyMacCas9 at the OsPDS-sgRNA09 (SM17) site.

(D) Genotypes of targeted T0 mutants by iSpyMacCas9 at the OsROC5-sgRNA03 (SM6) site. For all sequences, the PAM is highlighted in red and the protospacer is highlighted in blue.

(Chatterjee et al., 2020). While we were conducting this research, another group assessed SpyMacCas9 in rabbits and found that it preferred NAAA PAMs (Liu et al., 2019). Thus, all these studies concluded that iSpyMacCas9 favors A-rich PAMs. It is likely that the PAM preference identified for iSpyMacCas9 in rice would largely hold true for other plant species, although this requires future exploration. In our study, we found that iSpyMacCas9 was indeed more potent than SpyMacCas9, revealing the importance of the R221K and N394K mutations for improved activity (Spencer and Zhang, 2017). It is conceivable that protein engineering may be further used to relax iSpyMacCas9 PAM requirements as well as enhance its nuclease activity.

Base editors are powerful tools for introducing targeted single-nucleotide polymorphisms for crop improvement. However, for a given base editor, the base changes only occur within a defined editing window, which typically only spans a few nucleotides. Thus, it is very important to develop base editors that have altered PAM requirements as they would open up additional sites for editing. Here, we developed two iSpyMacCas9 CBE systems based on PmCDA1 and hAID cytidine deaminases. We found that they both resulted in efficient C to T base conversions at NAAR PAM sites, albeit with different base editing windows. Our data are consistent with previous reports on the use of PmCDA1 and hAID in plants for base editing (Shimatani et al., 2017; Ren et al., 2018; Tang et al., 2018). Interestingly, we found frequent C to G transversions in stable transgenic rice plants, but not in mesophyll protoplasts, at certain target sites with our PmCDA1-based iSpyMacCas9 CBE. This may reflect differences in DNA repair in different cell types. While the mechanism warrants further investigation, similar observations were previously reported by others (Nishida et al., 2016; Shimatani et al., 2017). We also developed one iSpyMacCas9 ABE system that resulted in efficient A to G base changes at NAAR PAM sites. ABE systems typically generate quite pure A to G edits without

Please cite this article in press as: Sretenovic et al., Expanding Plant Genome-Editing Scope by an Engineered iSpyMacCas9 System That Targets A-Rich PAM Sequences, Plant Communications (2020), https://doi.org/10.1016/j.xplc.2020.100101

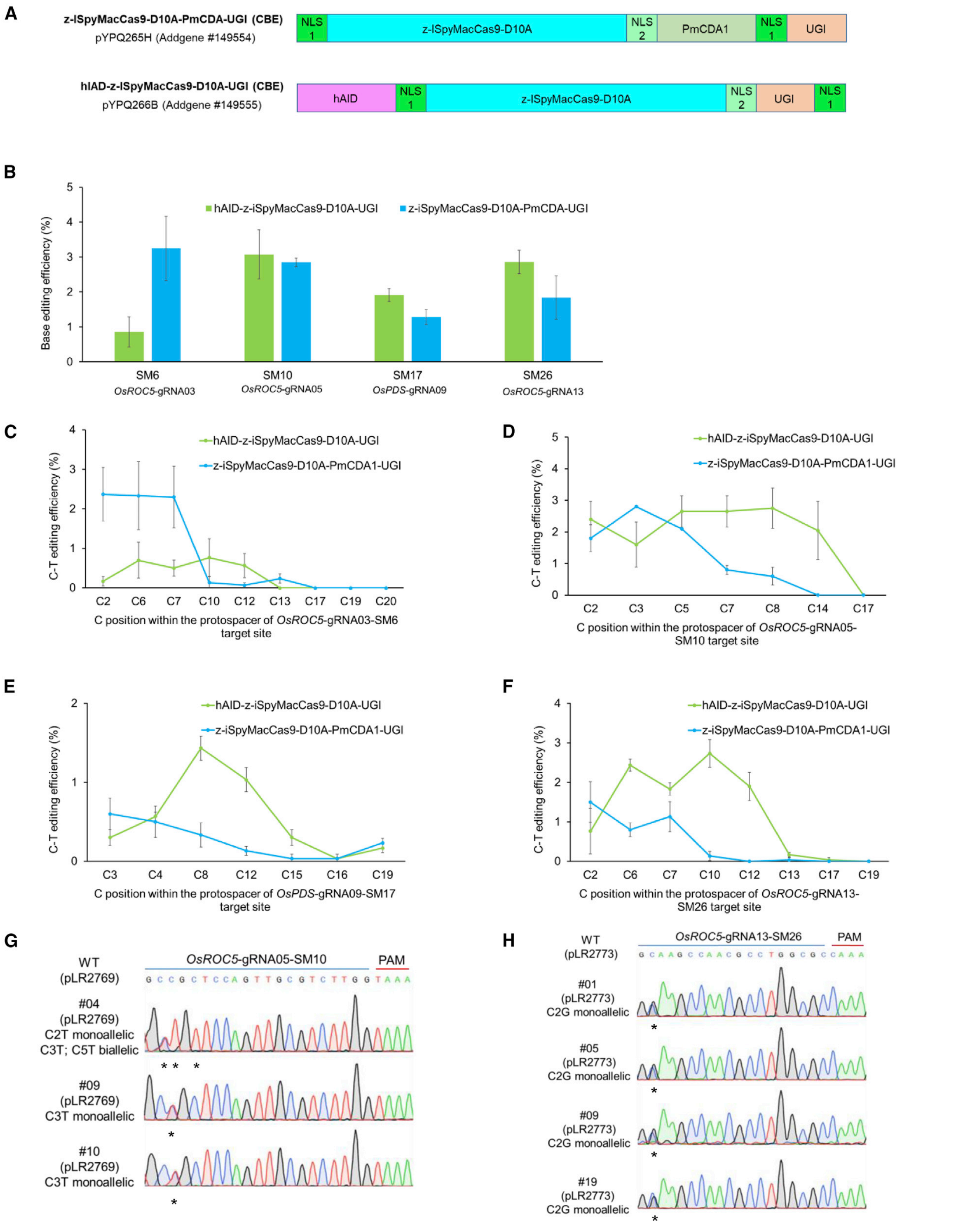

Figure 4. Development and Assessment of Two iSpyMacCas9 C to T Base Systems in Rice.
(A) Configurations of two cytidine base editors (CBEs) with PmCDA1 or hAID and iSpyMacCas9-D10A nickase.
(B) Assessment of editing efficiency of two iSpyMacCas9 CBEs at four target sites in rice protoplasts.
(C) Deamination window of PmCDA1 and hAID CBEs at the *OsROC5*-gRNA03 (SM6) target site.

(legend continued on next page)

Please cite this article in press as: Sretenovic et al., Expanding Plant Genome-Editing Scope by an Engineered iSpyMacCas9 System That Targets A-Rich PAM Sequences, Plant Communications (2020), https://doi.org/10.1016/j.xplc.2020.100101

Constructs	PAM Index	Targeted Rice Sites	Base Editor	Tested TO Lines	Mutated TO Lines (Number; Ratio)	Base-Edited TO Lines (Number; Ratio)	Indels in TO Lines (Number; Ratio)
pLR2769	TAAA	OsROC5-sg RNA05-SM10	ISpymac zCas9-PmCDA1-UGI	16	5; 31.3%	3; 18.8%	2; 12.5%
pLR2773	CAAA	OsROC5-sgRNA13-SM26	ISpymac zCasö-PmCDA1-UGI	18	9; 50%	7; 38.9%	5; 27.8%
pLR2771	TAAA	OsROC5-sgRNA05-SM10	wtTadA-TadA*-ISpymac zCas9	24	3; 12.5%	3; 12.5%	0; 0.0%

Table 2. Summary of Base Editing Efficiency of Two iSpyMacCas9 CBE Vectors and One iSpyMacCas9 ABE Vector in Rice T0 Lines.
UGI, uracil DNA glycosylase inhibitor.

inducing many indels (Kang et al., 2018; Li et al., 2018; Yan et al., 2018). The data generated from our iSpyMacCas9 ABE are consistent with these previous observations. Recently, the use of a single adenosine deaminase ecTadA*7.10 was shown to have improved base editing activity in rice (Hua et al., 2020b). It will be interesting to assess whether our iSpyMacCas9 ABE system can be further improved by using a similar architecture.

A unique advantage of our plant iSpyMacCas9 system over other existing CRISPR-Cas9 tools is its ability to target *cis*-regulatory elements that are typically A-T rich. For example, the quantitative trait variation in fruit size was generated by multiplexed promoter editing in tomatoes with the CRISPR-SpCas9 system (Rodriguez-Leal et al., 2017). Multiplexed editing of transcription activator-like effector binding sites in the promoters of *SWEET* genes in rice generated broad-spectrum disease resistance to bacterial blight (Oliva et al., 2019). The iSpyMacCas9 system uses the same sgRNA scaffold as the most popular SpCas9, making the system fully compatible with our previously developed multiplexed sgRNA expression systems (Lowder et al., 2015, 2017). Hence, the iSpyMacCas9 system can be easily applied for multiplexed promoter editing for crop trait engineering. In addition, iSpyMacCas9 base editors can be useful for introducing single-nucleotide polymorphisms in promoters to impact gene expression. At this point, this capability cannot be matched by A-T-rich PAM-targeting CRISPR-Cas12a and Cas12b systems as no base editors have been successfully developed in plants with these systems. Recently, SpCas9-based prime editors were demonstrated for precise genome editing in plants, although the editing efficiency was low (Hua et al., 2020a; Xu et al., 2020a, 2020b; Li et al., 2020; Lin et al., 2020; Tang et al., 2020). It will be useful to develop iSpyMacCas9 prime editors for directing specific changes at plant promoters to achieve desired gene expression. Finally, the A-rich PAM-targeting feature of iSpyMasCas9 also makes the system very appealing for transcriptional repression with CRISPR interference (CRISPRi) or transcriptional activation with CRISPR activation (CRISPRa) in plants. Previously, NGG PAMs targeting SpCas9 were predominantly used for CRISPRi (Piatek et al., 2014; Lowder et al., 2015) and CRISPRa (Lowder et al., 2015, 2018; Li et al., 2017b) in plants. By replacing SpCas9 with iSpyMacCas9 in such systems, more flexible promoter-targeting CRISPRi and CRISPRa systems could be engineered for plant applications.

In summary, we have developed an iSpyMacCas9 toolbox for plant genome editing at NAAR PAM sites. The four z-iSpyMacCas9 Gateway-compatible entry vectors that allow for targeted mutagenesis, C to T base editing, and A to G base editing have been deposited to Addgene for public dissemination. We envision that iSpyMacCas9 will enable the editing of A-T-rich *cis* elements for generating quantitative traits in addition to gene knockouts. Additionally, iSpyMacCas9 may facilitate other precise genome-editing applications, such as homology-directed repair and prime editing in plants. Finally, iSpyMacCas9 has the potential in developing CRISPRi and CRISPRa systems for transcriptional regulation in plants. These represent exciting fronts for future development and application of the iSpyMacCas9 systems.

MATERIALS AND METHODS

Genome-wide PAM Analysis in Rice, Maize, and Wheat

The genome of *O. sativa Nipponbare* (Oryza_sativa_nipponbare_v7.0_all.con) was downloaded from the Rice Genome Annotation Project (rice.plantbiology.msu.edu/pub/data/Eukaryotic_Projects/o_sativa/annotation_dbs/pseudomolecules/). The genomes of *T. aestivum* (Triticum_aestivum.IWGSC.dna.toplevel.fa) and *Z. mays* (Zea_mays.B73_RefGen_V4.dna.toplevel.fa) were downloaded from Ensembl (ftp://ftp.ensemblgenomes.org/pub/plants/release-45/fasta/triticum_aestivum/dna/, and ftp://ftp.ensemblgenomes.org/pub/plants/release-45/fasta/zea_mays/dna, respectively). The FASTA files were scanned line by line for the appropriate PAM patterns and their reverse complements using a regular expression search in Perl (version 5.26). For instance, to search for the positions of VTTV sites on a line, the WHILE loop (/(?=([ACG]TT[ACG])|([CGT]

(D) Deamination window of PmCDA1 and hAID CBEs at the *OsROC5*-gRNA05 (SM10) target site.
(E) Deamination window of PmCDA1 and hAID CBEs at the *OsPDS*-gRNA09 (SM17) target site.
(F) Deamination window of PmCDA1 and hAID CBEs at the *OsROC5*-gRNA13 (SM26) target site.
(G) Independent T0 lines with clean C to T base editing at the OsROC5-sgRNA05 site. Only three out of five such lines are shown. Base changes are indicated by asterisks.
(H) Independent T0 lines with clean C to G base editing at the OsROC5-sgRNA13 site. Base changes are indicated by asterisks. Error bars represent standard deviations of three independent experiments.

Please cite this article in press as: Sretenovic et al., Expanding Plant Genome-Editing Scope by an Engineered iSpyMacCas9 System That Targets A-Rich PAM Sequences, Plant Communications (2020), https://doi.org/10.1016/j.xplc.2020.100101

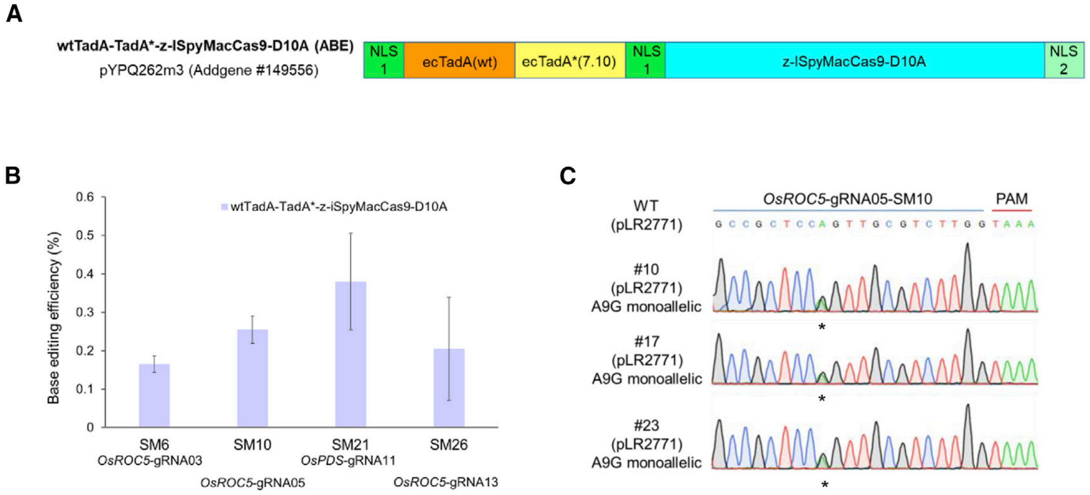

Figure 5. Development and Assessment of One iSpyMacCas9 A to G Base System in Rice.

(A) Configurations of the adenine base editor based on iSpyMacCas9-D10A nickase.

(B) Assessment of base editing efficiency of the iSpyMacCas9 ABE at four target sites in rice protoplasts. Error bars represent standard deviations of three biological replicates.

(C) Independent T0 lines with clean A to G base editing at the *OsROC5*-sgRNA05 (SM10) site. Base changes are indicated by asterisks.

AA[CGT]))/g) was utilized along with proper coding to handle patterns that break over two lines. All PAM sites were tabulated by the genomic position along with the flanking sequence for post-processing and analyses, for instance, to obtain distributions of PAM site spacings, and, with appropriate annotation files, their abundance in relevant genomic features, such as open reading frames and non-coding regions.

Vector Construction

To prepare plant codon optimized (pco) and maize codon optimized (z) SpyMacCas9 attL1-attR5 Gateway-compatible entry clones pYPQ150-SpyMac and pYPQ166-SpyMac, *Arabidopsis thaliana* and maize codon-optimized PI domains from *Streptococcus macacae* (Chatterjee et al., 2020) were synthesized (IDT gBlocks). pYPQ150 (Addgene no. 69301) (Lowder et al., 2015) containing pco-SpyCas9 was enzymatically digested with XmnI (NEB, catalog no. R0194*) and AatII (NEB, catalog no. R0117*). pYPQ166 (Addgene no. 109328) (Lowder et al., 2015) containing z-SpyCas9 was enzymatically digested with MscI (NEB, catalog no. R0534*) and SacI (NEB, catalog no. R3156*). Based on the synthesized gBlocks and digested vectors, the NEBuilder HiFi DNA assembly kit was used to replace the *Streptococcus pyogenes* Cas9 PI domain in vectors pYPQ150 and pYPQ166 to obtain pYPQ150-SpyMac and pYPQ166-SpyMac, respectively. Mutations R221K and N394K (Chatterjee et al., 2020) were introduced in pYPQ150-SpyMac and pYPQ166-Spymac with mismatched primers 150-R221K-F, 150-R221K-R; 150-N394K-F, 150-N394K-R; 166-R221K-F, 166-R221K-R; 166-N394K-F, 166-N394K-R (Supplemental Table 1). The NEB Q5 mutagenesis kit was used to prepare improved SpyMacCas9 attL1-attR5 Gateway-compatible entry clones pYPQ150-iSpyMac and pYPQ166-iSpyMac (Addgene no. 149553). z-iSpyMacCas9-derived attL1-attR5 Gateway-compatible base editors pYPQ265H (Addgene no. 149554), pYPQ266B (Addgene no. 149555), and pYPQ262m3 (Addgene no. 149556) were prepared using the NEBuilder HiFi DNA assembly kit with primers ISM-Ins_F, ISM-Ins_R, zISMBE-BB_F, and zISMBE-BB_R from vectors containing PmCDA1 (Tang et al., 2018), hAID (Ren et al., 2018), and ecTadA(wt)-exTadA*(7.10) (Koblan et al., 2018) deaminases.

For the design of the sgRNAs, the Nipponbare reference sequence of genes *OsPDS* and *OsROC5* was used. Using Phytozome V12.1 genomic data (https://phytozome.jgi.doe.gov/pz/portal.html), we validated that the Nipponbare reference sequences for both aforementioned genes were 100% identical in Kitaake, a rice variety used in this study. T-DNA vectors (Supplemental Table 2) for CRISPR-Cas9 were constructed using Gateway LR assembly reactions based on the protocols described previously (Lowder et al., 2015). In brief, forward and reverse primers (Supplemental Table 1) for sgRNAs were phosphorylated with T4 polynucleotide kinase (NEB, catalog no. M0201*), annealed, and ligated with T4 DNA ligase (NEB, catalog no. M0202*) into BsmBI (Thermo Fisher Scientific, catalog no. ER045*) restriction-digested pYPQ141C (Addgene no. 69292) or pYPQ141D (Addgene no. 69293) sgRNA entry clones. Individual Gateway LR reactions consisted of the attL5-attL2 sgRNA entry clone, attL1-attR5 (i)SpyMacCas9 entry clone, and attR1-attR2 destination vector pYPQ203 (Addgene no. 86 207) containing a ZmUbi1 promoter for Cas9 expression. Both sgRNA and Cas9 entry clone recombination regions were confirmed by Sanger sequencing. The final T-DNA vectors were confirmed by restriction digestion with EcoRI (NEB, catalog no. R0101*).

Rice Protoplast Isolation and Transformation

The Japonica cultivar *Kitaake* was used in this study. The rice seedlings were grown on 1/2 MS solid medium for 12–14 days in the dark at 28°C. Rice protoplast extraction and transformation were performed according to our previously published protocols (Lowder et al., 2015; Tang et al., 2017). In brief, healthy leaves were cut into 0.5- to 1.0-mm strips and transferred into the enzyme solution, followed by vacuum-infiltration for 30 min and incubation at 70–80 rpm for 8 h at 25°C in the dark. Each digestion mixture was filtered through a 40-μm cell strainer. After washing twice with a W5 washing buffer, protoplasts were examined and counted under a microscope. The final protoplast concentration was adjusted to 2×10^6 per ml. For protoplast transformation, 30 μg of plasmid DNA in 30 μl (1 μg/μl; prepared using a QIAGEN Midiprep kit) was used to transform 200 μl of protoplasts by gently mixing with 230 μl of 40% PEG transformation buffer. After incubation for 30 min in darkness, the reactions were stopped by adding 900 μl of W5 washing buffer. The protoplasts were centrifuged and transferred into a 12-well culture plate and incubated at 32°C in darkness for 48 h.

Validation of Targeted Mutagenesis in Rice Protoplasts

Two targets associated with each of 16 possible NAAN PAMs were chosen based on the presence of a type II restriction enzyme site

Please cite this article in press as: Sretenovic et al., Expanding Plant Genome-Editing Scope by an Engineered iSpyMacCas9 System That Targets A-Rich PAM Sequences, Plant Communications (2020), https://doi.org/10.1016/j.xplc.2020.100101

superimposed at the cleavage sites, allowing for the validation of target site mutations with restriction fragment length polymorphism assays. Genomic DNA was extracted from the protoplasts after 48 h of incubation in the dark at 32°C. Target sites were PCR amplified (primers listed in Supplemental Table 1), digested, and run on 2% gels. ImageJ 1.52p computer software was used to obtain the intensities of bands representing uncut and cut DNA fragments. Genome-editing efficiency was calculated as the ratio of uncut bands against all (cut and uncut) bands. Positive control (cut WT target site) and negative control (uncut WT target site) were carried out for system calibration.

Validation of Base Editing in Rice Protoplasts

The NGS of PCR amplicons was used for the detection and quantification of base editing mutations at the target sites. Genomic DNA was extracted from the protoplasts after 48 h of incubation in the dark at 32°C. Using protoplast DNA as the template, genome regions of targeted sites were amplified with barcoded primers (Supplemental Table 1) according to our previously published protocols (Zhong et al., 2019). The specificity of PCR reactions was verified by gel electrophoresis. Transcripts were column-purified, pooled, and sequenced by GENEWIZ (NJ, USA) with the Illumina MiSeq sequencing platform. The raw data were analyzed using BE-Analyzer online software (Hwang et al., 2018).

Stable Transformation of Rice

The Japonica cultivar *Kitaake* was used for rice stable transformation. *Agrobacterium*-mediated transformation was carried out by following a transformation protocol previously established in the lab (Lowder et al., 2015).

Validation of Genetic Modifications in T0 Plants

Sanger sequencing was used to determine genetic modifications at the target sites in T0 plants. Genomic DNA was extracted from the protoplasts after 48 h of incubation in the dark at 32°C. Using T0 genomic DNA as the template, the genome regions of targeted sites were amplified with primers (Supplemental Table 1). The specificity of PCR reactions was verified by gel electrophoresis. PCR reactions were enzymatically cleaned with the ExoSAP kit (NEB) and Sanger sequenced by GENEWIZ. The raw data were analyzed with SnapGene software.

SUPPLEMENTAL INFORMATION

Supplemental Information can be found online at *Plant Communications Online*.

FUNDING

This work was supported by startup funds from the University of Maryland, the National Science Foundation Plant Genome Research Program grant (award no. IOS-1758745), and the Biotechnology Risk Assessment Grant Program competitive grant (award no. 2018-33522-28789) from the U.S. Department of Agriculture.

AUTHOR CONTRIBUTIONS

Y.Q. conceived and designed the experiments. S.S. designed the constructs. S.S., D.Y., and A.L. generated the cloning and expression vectors. S.S. and D.Y. conducted rice protoplast and stable transformation and analysis. S.S performed NGS sample preparation and data analysis. J.D.S. and S.M.M. performed the genome-wide PAM analyses in rice, maize, and wheat. S.S. and Y.Q. analyzed all the data and wrote the manuscript. All authors participated in the discussion and revision of the manuscript.

ACKNOWLEDGMENTS

The authors thank Noah Jakimo and Joseph Jacobson at the MIT Media Lab for providing SpyMacCas9 and iSpyMacCas9 protein sequences. The authors also thank Addgene for disseminating our iSpyMacCas9 plant genome-editing vectors. No conflict of interest declared.

Received: June 5, 2020
Revised: July 12, 2020
Accepted: July 20, 2020
Published: ■■ ■■, ■■

REFERENCES

Chatterjee, P., Lee, J., Nip, L., Koseki, S.R.T., Tysinger, E., Sontheimer, E.J., Jacobson, J.M., and Jakimo, N. (2020). A Cas9 with PAM recognition for adenine dinucleotides. Nat. Commun. **11**:2474.

Eid, A., Alshareef, S., and Mahfouz, M.M. (2018). CRISPR base editors: genome editing without double-stranded breaks. Biochem. J. **475**:1955–1964.

Endo, M., Mikami, M., Endo, A., Kaya, H., Itoh, T., Nishimasu, H., Nureki, O., and Toki, S. (2019). Genome editing in plants by engineered CRISPR-Cas9 recognizing NG PAM. Nat. Plants **5**:14–17.

Garneau, J.E., Dupuis, M.E., Villion, M., Romero, D.A., Barrangou, R., Boyaval, P., Fremaux, C., Horvath, P., Magadan, A.H., and Moineau, S. (2010). The CRISPR/Cas bacterial immune system cleaves bacteriophage and plasmid DNA. Nature **468**:67–71.

Gaudelli, N.M., Komor, A.C., Rees, H.A., Packer, M.S., Badran, A.H., Bryson, D.I., and Liu, D.R. (2017). Programmable base editing of A*T to G*C in genomic DNA without DNA cleavage. Nature **551**:464–471.

Ge, Z., Zheng, L., Zhao, Y., Jiang, J., Zhang, E.J., Liu, T., Gu, H., and Qu, L.J. (2019). Engineered xCas9 and SpCas9-NG variants broaden PAM recognition sites to generate mutations in *Arabidopsis* plants. Plant Biotechnol. J. **17**:1865–1867.

Gurel, F., Zhang, Y., Sretenovic, S., and Qi, Y. (2020). CRISPR-Cas nucleases and base editors for plant genome editing. aBiotech, 1–14. https://doi.org/10.1007/s42994-019-00010-0.

Hu, J.H., Miller, S.M., Geurts, M.H., Tang, W., Chen, L., Sun, N., Zeina, C.M., Gao, X., Rees, H.A., Lin, Z., et al. (2018a). Evolved Cas9 variants with broad PAM compatibility and high DNA specificity. Nature **556**:57–63.

Hu, X., Meng, X., Liu, Q., Li, J., and Wang, K. (2018b). Increasing the efficiency of CRISPR-Cas9-VQR precise genome editing in rice. Plant Biotechnol. J. **16**:292–297.

Hu, X., Wang, C., Fu, Y., Liu, Q., Jiao, X., and Wang, K. (2016). Expanding the range of CRISPR/Cas9 genome editing in rice. Mol. Plant **9**:943–945.

Hua, K., Jiang, Y., Tao, X., and Zhu, J.K. (2020a). Precision genome engineering in rice using prime editing system. Plant Biotechnol. J. https://doi.org/10.1111/pbi.13395.

Hua, K., Tao, X., Han, P., Wang, R., and Zhu, J.K. (2019). Genome engineering in rice using Cas9 variants that recognize NG PAM sequences. Mol. Plant **12**:1003–1014.

Hua, K., Tao, X., Liang, W., Zhang, Z., Gou, R., and Zhu, J.K. (2020b). Simplified adenine base editors improve adenine base editing efficiency in rice. Plant Biotechnol. J. **18**:770–778.

Hwang, G.H., Park, J., Lim, K., et al. (2018). Web-based design and analysis tools for CRISPR base editing. BMC Bioinformatics **19**:542.

Jia, H., Xu, J., Orbovic, V., Zhang, Y., and Wang, N. (2017). Editing citrus genome via SaCas9/sgRNA system. Front Plant Sci. **8**:2135.

Jinek, M., Chylinski, K., Fonfara, I., Hauer, M., Doudna, J.A., and Charpentier, E. (2012). A programmable dual-RNA-guided DNA endonuclease in adaptive bacterial immunity. Science **337**:816–821.

Kang, B.C., Yun, J.Y., Kim, S.T., Shin, Y., Ryu, J., Choi, M., Woo, J.W., and Kim, J.S. (2018). Precision genome engineering through adenine base editing in plants. Nat. Plants **4**:427–431.

Please cite this article in press as: Sretenovic et al., Expanding Plant Genome-Editing Scope by an Engineered iSpyMacCas9 System That Targets A-Rich PAM Sequences, Plant Communications (2020), https://doi.org/10.1016/j.xplc.2020.100101

Kaya, H., Ishibashi, K., and Toki, S. (2017). A split *Staphylococcus aureus* Cas9 as a compact genome editing tool in plants. Plant Cell Physiol. **58**:643–649.

Kaya, H., Mikami, M., Endo, A., Endo, M., and Toki, S. (2016). Highly specific targeted mutagenesis in plants using *Staphylococcus aureus* Cas9. Sci. Rep. **6**:26871.

Kleinstiver, B.P., Prew, M.S., Tsai, S.Q., Topkar, V.V., Nguyen, N.T., Zheng, Z., Gonzales, A.P., Li, Z., Peterson, R.T., Yeh, J.R., et al. (2015). Engineered CRISPR-Cas9 nucleases with altered PAM specificities. Nature **523**:481–485.

Koblan, L.W., Doman, J.L., Wilson, C., Levy, J.M., Tay, T., Newby, G.A., Maianti, J.P., Raguram, A., and Liu, D.R. (2018). Improving cytidine and adenine base editors by expression optimization and ancestral reconstruction. Nat. Biotechnol. **36**:843–846.

Komor, A.C., Kim, Y.B., Packer, M.S., Zuris, J.A., and Liu, D.R. (2016). Programmable editing of a target base in genomic DNA without double-stranded DNA cleavage. Nature **533**:420–424.

Li, C., Zong, Y., Wang, Y., Jin, S., Zhang, D., Song, Q., Zhang, R., and Gao, C. (2018). Expanded base editing in rice and wheat using a Cas9-adenosine deaminase fusion. Genome Biol. **19**:59.

Li, H., Li, J., Chen, J., Yan, L., and Xia, L. (2020). Precise modifications of both exogenous and endogenous genes in rice by prime editing. Mol. Plant **13**:671–674.

Li, J., Luo, J., Xu, M., Li, S., Zhang, J., Li, H., Yan, L., Zhao, Y., and Xia, L. (2019a). Plant genome editing using xCas9 with expanded PAM compatibility. J. Genet. Genomics **46**:277–280.

Li, J., Sun, Y., Du, J., Zhao, Y., and Xia, L. (2017a). Generation of targeted point mutations in rice by a modified CRISPR/Cas9 system. Mol. Plant **10**:526–529.

Li, J.F., Norville, J.E., Aach, J., McCormack, M., Zhang, D., Bush, J., Church, G.M., and Sheen, J. (2013). Multiplex and homologous recombination-mediated genome editing in *Arabidopsis* and *Nicotiana benthamiana* using guide RNA and Cas9. Nat. Biotechnol. **31**:688–691.

Li, Z., Xiong, X., Wang, F., Liang, J., and Li, J.F. (2019b). Gene disruption through base editing-induced messenger RNA missplicing in plants. New Phytol. **222**:1139–1148.

Li, Z., Zhang, D., Xiong, X., Yan, B., Xie, W., Sheen, J., and Li, J.F. (2017b). A potent Cas9-derived gene activator for plant and mammalian cells. Nat. Plants **3**:930–936.

Lin, Q., Zong, Y., Xue, C., Wang, S., Jin, S., Zhu, Z., Wang, Y., Anzalone, A.V., Raguram, A., Doman, J.L., et al. (2020). Prime genome editing in rice and wheat. Nat. Biotechnol. **38**:582–585.

Liu, Z., Shan, H., Chen, S., Chen, M., Song, Y., Lai, L., and Li, Z. (2019). Efficient base editing with expanded targeting scope using an engineered Spy-mac Cas9 variant. Cell Discov. **5**:58.

Lowder, L., Malzahn, A., and Qi, Y. (2017). Rapid construction of multiplexed CRISPR-Cas9 systems for plant genome editing. Methods Mol. Biol. **1578**:291–307.

Lowder, L.G., Zhang, D., Baltes, N.J., Paul, J.W., Tang, X., Zheng, X., Voytas, D.F., Hsieh, T.F., Zhang, Y., and Qi, Y. (2015). A CRISPR/Cas9 toolbox for multiplexed plant genome editing and transcriptional regulation. Plant Physiol. **169**:971–985.

Lowder, L.G., Zhou, J., Zhang, Y., Malzahn, A., Zhong, Z., Hsieh, T.F., Voytas, D.F., Zhang, Y., and Qi, Y. (2018). Robust transcriptional activation in plants using multiplexed CRISPR-Act2.0 and mTALE-Act systems. Mol. Plant **11**:245–256.

Lu, Y., and Zhu, J.K. (2017). Precise editing of a target base in the rice genome using a modified CRISPR/Cas9 system. Mol. Plant **10**:523–525.

Miki, D., and Shimamoto, K. (2004). Simple RNAi vectors for stable and transient suppression of gene function in rice. Plant Cell Physiol. **45**:490–495.

Molla, K.A., Shih, J., and Yang, Y. (2020). Single-nucleotide editing for zebra3 and wsl5 phenotypes in rice using CRISPR/Cas9-mediated adenine base. aBiotech **1**:106–118.

Molla, K.A., and Yang, Y. (2019). CRISPR/Cas-mediated base editing: technical considerations and practical applications. Trends Biotechnol. **37**:1121–1142.

Muller, M., Lee, C.M., Gasiunas, G., Davis, T.H., Cradick, T.J., Siksnys, V., Bao, G., Cathomen, T., and Mussolino, C. (2016). *Streptococcus thermophilus* CRISPR-Cas9 systems enable specific editing of the human genome. Mol. Ther. **24**:636–644.

Negishi, K., Kaya, H., Abe, K., Hara, N., Saika, H., and Toki, S. (2019). An adenine base editor with expanded targeting scope using SpCas9-NGv1 in rice. Plant Biotechnol. J. **17**:1476–1478.

Nishida, K., Arazoe, T., Yachie, N., Banno, S., Kakimoto, M., Tabata, M., Mochizuki, M., Miyabe, A., Araki, M., Hara, K.Y., et al. (2016). Targeted nucleotide editing using hybrid prokaryotic and vertebrate adaptive immune systems. Science **353**:aaf8729.

Nishimasu, H., Shi, X., Ishiguro, S., Gao, L., Hirano, S., Okazaki, S., Noda, T., Abudayyeh, O.O., Gootenberg, J.S., Mori, H., et al. (2018). Engineered CRISPR-Cas9 nuclease with expanded targeting space. Science **361**:1259–1262.

Oliva, R., Ji, C., Atienza-Grande, G., Huguet-Tapia, J.C., Perez-Quintero, A., Li, T., Eom, J.S., Li, C., Nguyen, H., Liu, B., et al. (2019). Broad-spectrum resistance to bacterial blight in rice using genome editing. Nat. Biotechnol. **37**:1344–1350.

Piatek, A., Ali, Z., Baazim, H., Li, L., Abulfaraj, A., Al-Shareef, S., Aouida, M., and Mahfouz, M.M. (2014). RNA-guided transcriptional regulation in planta via synthetic dCas9-based transcription factors. Plant Biotechnol. J. **13**:578–589.

Puchta, H. (2005). The repair of double-strand breaks in plants: mechanisms and consequences for genome evolution. J. Exp. Bot. **56**:1–14.

Ran, F.A., Cong, L., Yan, W.X., Scott, D.A., Gootenberg, J.S., Kriz, A.J., Zetsche, B., Shalem, O., Wu, X., Makarova, K.S., et al. (2015). In vivo genome editing using *Staphylococcus aureus* Cas9. Nature **520**:186–191.

Ren, B., Liu, L., Li, S., Kuang, Y., Wang, J., Zhang, D., Zhou, X., Lin, H., and Zhou, H. (2019). Cas9-NG greatly expands the targeting scope of the genome-editing toolkit by recognizing NG and other atypical PAMs in rice. Mol. Plant **12**:1015–1026.

Ren, B., Yan, F., Kuang, Y., Li, N., Zhang, D., Lin, H., and Zhou, H. (2017). A CRISPR/Cas9 toolkit for efficient targeted base editing to induce genetic variations in rice. Sci. China Life Sci. **60**:516–519.

Ren, B., Yan, F., Kuang, Y., Li, N., Zhang, D., Zhou, X., Lin, H., and Zhou, H. (2018). Improved base editor for efficiently inducing genetic variations in rice with CRISPR/Cas9-guided hyperactive hAID mutant. Mol. Plant **11**:623–626.

Rodriguez-Leal, D., Lemmon, Z.H., Man, J., Bartlett, M.E., and Lippman, Z.B. (2017). Engineering quantitative trait variation for crop improvement by genome editing. Cell **171**:470–480 e478.

Shimatani, Z., Kashojiya, S., Takayama, M., Terada, R., Arazoe, T., Ishii, H., Teramura, H., Yamamoto, T., Komatsu, H., Miura, K., et al. (2017). Targeted base editing in rice and tomato using a CRISPR-Cas9 cytidine deaminase fusion. Nat. Biotechnol. **35**:441–443.

Spencer, J.M., and Zhang, X. (2017). Deep mutational scanning of *S. pyogenes* Cas9 reveals important functional domains. Sci. Rep. **7**:16836.

Please cite this article in press as: Sretenovic et al., Expanding Plant Genome-Editing Scope by an Engineered iSpyMacCas9 System That Targets A-Rich PAM Sequences, Plant Communications (2020), https://doi.org/10.1016/j.xplc.2020.100101

Steinert, J., Schiml, S., Fauser, F., and Puchta, H. (2015). Highly efficient heritable plant genome engineering using Cas9 orthologues from Streptococcus thermophilus and Staphylococcus aureus. Plant J **84**:1295–1305.

Tang, X., Lowder, L.G., Zhang, T., et al. (2017). A CRISPR-Cpf1 system for efficient genome editing and transcriptional repression in plants. Nat. Plants **3**:17018.

Tang, X., Ren, Q., Yang, L., Bao, Y., Zhong, Z., He, Y., Liu, S., Qi, C., Liu, B., Wang, Y., et al. (2018). Single transcript unit CRISPR 2.0 systems for robust Cas9 and Cas12a mediated plant genome editing. Plant Biotechnol. J. **17**:1431–1445.

Tang, X., Sretenovic, S., Ren, Q., Jia, X., Li, M., Fan, T., Yin, D., Xiang, S., Guo, Y., Liu, L., et al. (2020). Plant prime editors enable precise gene editing in rice cells. Mol. Plant **13**:667–670.

Wang, J., Meng, X., Hu, X., Sun, T., Li, J., Wang, K., and Yu, H. (2018). xCas9 expands the scope of genome editing with reduced efficiency in rice. Plant Biotechnol. J. **17**:709–711.

Xing, H.L., Dong, L., Wang, Z.P., Zhang, H.Y., Han, C.Y., Liu, B., Wang, X.C., and Chen, Q.J. (2014). A CRISPR/Cas9 toolkit for multiplex genome editing in plants. BMC Plant Biol. **14**:327.

Xu, R., Li, J., Liu, X., Shan, T., Qin, R., and Wei, P. (2020a). Development of plant prime-editing systems for precise genome editing. Plant Commun. https://doi.org/10.1016/j.xplc.2020.100043.

Xu, W., Zhang, C., Yang, Y., Zhao, S., Kang, G., He, X., Song, J., and Yang, J. (2020b). Versatile nucleotides substitution in plant using an improved prime editing system. Mol. Plant **13**:675–678.

Xue, C., Zhang, H., Lin, Q., Fan, R., and Gao, C. (2018). Manipulating mRNA splicing by base editing in plants. Sci. China Life Sci. **61**:1293–1300.

Yan, F., Kuang, Y., Ren, B., Wang, J., Zhang, D., Lin, H., Yang, B., Zhou, X., and Zhou, H. (2018). Highly efficient A.T to G.C base editing by Cas9n-guided tRNA adenosine deaminase in rice. Mol. Plant **11**:631–634.

Zeng, D., Li, X., Huang, J., Li, Y., Cai, S., Yu, W., Li, Y., Huang, Y., Xie, X., Gong, Q., et al. (2020). Engineered Cas9 variant tools expand targeting scope of genome and base editing in rice. Plant Biotechnol. J. **18**:1348–1350.

Zhang, Y., Malzahn, A.A., Sretenovic, S., and Qi, Y. (2019). The emerging and uncultivated potential of CRISPR technology in plant science. Nat. Plants **5**:778–794.

Zhong, Z., Sretenovic, S., Ren, Q., Yang, L., Bao, Y., Qi, C., Yuan, M., He, Y., Liu, S., Liu, X., et al. (2019). Improving plant genome editing with high-fidelity xCas9 and non-canonical PAM-targeting Cas9-NG. Mol. Plant **12**:1027–1036.

Zong, Y., Song, Q., Li, C., Jin, S., Zhang, D., Wang, Y., Qiu, J.L., and Gao, C. (2018). Efficient C-to-T base editing in plants using a fusion of nCas9 and human APOBEC3A. Nat. Biotechnol. **36**:950–953.

Zong, Y., Wang, Y., Li, C., Zhang, R., Chen, K., Ran, Y., Qiu, J.L., Wang, D., and Gao, C. (2017). Precise base editing in rice, wheat and maize with a Cas9-cytidine deaminase fusion. Nat. Biotechnol. **35**:438–440.

Zhong, Z., Zhang, Y., You, Q., et al. (2018). Plant genome editing using FnCpf1 and LbCpf1 nucleases at redefined and altered PAM sites. Mol. Plant **11**:999–1002.

Zou, L.P., Sun, X.H., Zhang, Z.G., Liu, P., Wu, J.X., Tian, C.J., Qiu, J.L., and Lu, T.G. (2011). Leaf rolling controlled by the homeodomain leucine zipper class IV gene Roc5 in rice. Plant Physiol. **156**:1589–1602.

Plant Communications, Volume *1*

Supplemental Information

Expanding Plant Genome-Editing Scope by an Engineered iSpyMac-Cas9 System That Targets A-Rich PAM Sequences

Simon Sretenovic, Desuo Yin, Adam Levav, Jeremy D. Selengut, Stephen M. Mount, and Yiping Qi

Expanding Plant Genome Editing Scope by An Engineered iSpyMacCas9 System Targeting the A-rich PAM Sequences

Simon Sretenovic[1, ⱡ], Desuo Yin[1,2, ⱡ], Adam Levav[1,3], Jeremy D. Selengut[4], Stephen M. Mount[5], Yiping Qi[1,6, *]

[1]Department of Plant Science and Landscape Architecture, University of Maryland, College Park, Maryland 20742, USA; [2]Hubei Academy of Agricultural Sciences, Wuhan 430064, China; [3]Montgomery Blair High School, Silver Spring, Maryland 20901, USA; [4]Center for Bioinformatics and Computational Biology, University of Maryland, College Park, Maryland 20742, USA; [5]Department of Cell Biology and Molecular Genetics, University of Maryland, College Park, Maryland 20742, USA; [6]Institute for Bioscience and Biotechnology Research, University of Maryland, Rockville, Maryland 20850, USA.

ⱡ These authors contributed equally to this work.

***Corresponding author**

Yiping Qi, Department of Plant Science and Landscape Architecture, University of Maryland, College Park, MD 20742, USA; Email: Yiping@umd.edu

SUPPLEMENTAL INFORMATION

- Supplemental Table 1. Oligos used in this study.
- Supplemental Table 2. T-DNA expression constructs used in this study.
- Supplemental Figure 1. Raw RFLP data of testing 4 (i)SpyMacCas9 variants at 4 target sites in 3 replicates.
- Supplemental Figure 2: Raw RFLP data of testing z-iSpyMacCas9 at 28 target sites in 3 replicates.
- Supplemental Figure 3. Mutated T0 lines induced by SpyMacCas9 and iSpyMacCas9 with OsPDS-sgRNA11.
- Supplemental Figure 4. Indel mutants generated by PmCDA1 based iSpyMacCas9 CBE with OsROC5-sgRNA05.

Supplemental Table 1. Oligos used in this study.

Name	Sequence 5'-3'	Description
AAAA-PDS-gR1-F	tggcATGAGTAGGAAGTTGTCCCGC	Forward primer to prepare gRNA
AAAA-PDS-gR1-R	aaacGCGGGACAACTTCCTACTCAT	Reverse primer to prepare gRNA
AAAA-ROC5-gR1-F	GTGTgctccagttgcgtcttggt	Forward primer to prepare gRNA
AAAA-ROC5-gR1-R	AAACaccaagacgcaactggagc	Reverse primer to prepare gRNA
AAAT-PDS-gR2-F	TGGCatagcacaacgagaaccttaa	Forward primer to prepare gRNA
AAAT-PDS-gR2-R	AAACttaaggttctcgttgtgctat	Reverse primer to prepare gRNA
AAAT-ROC5-gR2-F	TGGCagctgcttacaaaaccggag	Forward primer to prepare gRNA
AAAT-ROC5-gR2-R	AAACctccggttttgtaagcagct	Reverse primer to prepare gRNA
AAAG-PDS-gR3-F	tggcAGTAGGAAGTTGTCCCGCA	Forward primer to prepare gRNA
AAAG-PDS-gR3-R	aaacTGCGGGACAACTTCCTACT	Reverse primer to prepare gRNA
AAAG-ROC5-gR3-F	GTGTgcaagccaacgcctggcgcc	Forward primer to prepare gRNA
AAAG-ROC5-gR3-R	AAACggcgccaggcgttggcttgc	Reverse primer to prepare gRNA
AAAC-PDS-gR4-F	tggcAGGTCTTGGAAAGTCCTGGC	Forward primer to prepare gRNA
AAAC-PDS-gR4-R	aaacGCCAGGACTTTCCAAGACCT	Reverse primer to prepare gRNA
AAAC-ROC5-gR4-F	GTGTgctccagttgcgtcttggta	Forward primer to prepare gRNA
AAAC-ROC5-gR4-R	AAACtaccaagacgcaactggagc	Reverse primer to prepare gRNA
TAAA-PDS-gR5-F	tggcAAATCTCTTGTCTTAAGGAA	Forward primer to prepare gRNA
TAAA-PDS-gR5-R	aaacTTCCTTAAGACAAGAGATTT	Reverse primer to prepare gRNA
TAAA-ROC5-gR5-F	GTGTgccgctccagttgcgtcttgg	Forward primer to prepare gRNA
TAAA-ROC5-gR5-R	AAACccaagacgcaactggagcggc	Reverse primer to prepare gRNA
TAAT-PDS-gR6-F	GTGTgtcatgtgtgttgcatatgag	Forward primer to prepare gRNA
TAAT-PDS-gR6-R	AAACctcatatgcaacacacatgac	Reverse primer to prepare gRNA
TAAT-ROC5-gR6-F	TGGCatgcatcaaatgcaacacg	Forward primer to prepare gRNA
TAAT-ROC5-gR6-R	AAACcgtgttgcatttgatgcat	Reverse primer to prepare gRNA
TAAG-PDS-gR7-F	gtgtGGAGTGAAATCTCTTGTCT	Forward primer to prepare gRNA
TAAG-PDS-gR7-R	aaacAGACAAGAGATTTCACTCC	Reverse primer to prepare gRNA
TAAG-ROC5-gR7-F	GTGTgttacatttctccggttttg	Forward primer to prepare gRNA
TAAG-ROC5-gR7-R	AAACcaaaaccggagaaatgtaac	Reverse primer to prepare gRNA
TAAC-PDS-gR8-F	tggcATGGTCAACAATAGGCATGCA	Forward primer to prepare gRNA
TAAC-PDS-gR8-R	aaacTGCATGCCTATTGTTGACCAT	Reverse primer to prepare gRNA
TAAC-ROC5-gR8-F	TGGCActctgtgaaatgaatgcaag	Forward primer to prepare gRNA
TAAC-ROC5-gR8-R	AAACcttgcattcatttcacagagT	Reverse primer to prepare gRNA
GAAA-PDS-gR9-F	gtgtGTCCTGGCAAACAACctgca	Forward primer to prepare gRNA
GAAA-PDS-gR9-R	AAACtgcagGTTGTTTGCCAGGAC	Reverse primer to prepare gRNA
GAAA-ROC5-gR9-F	TGGCatgtcagaccaccgtctctgt	Forward primer to prepare gRNA
GAAA-ROC5-gR9-R	AAACacagagacggtggtctgacat	Reverse primer to prepare gRNA
GAAT-PDS-gR10-F	gtgtGTGAAATCTCTTGTCTTAAG	Forward primer to prepare gRNA
GAAT-PDS-gR10-R	aaacCTTAAGACAAGAGATTTCAC	Reverse primer to prepare gRNA
GAAT-ROC5-gR10-F	TGGCacctgtgggtgccgttcca	Forward primer to prepare gRNA
GAAT-ROC5-gR10-R	AAACtggaacggcacccacaggt	Reverse primer to prepare gRNA
GAAG-PDS-gR11-F	gtgtGCCTGAAAAGTCTGCAGTCT	Forward primer to prepare gRNA
GAAG-PDS-gR11-R	aaacAGACTGCAGACTTTTCAGGC	Reverse primer to prepare gRNA
GAAG-ROC5-gR11-F	GTGTgcttgtcgttctcctgcttga	Forward primer to prepare gRNA
GAAG-ROC5-gR11-R	AAACtcaagcaggagaacgacaagc	Reverse primer to prepare gRNA
GAAC-PDS-gR12-F	GTGTgtaaggtactactccggaaa	Forward primer to prepare gRNA
GAAC-PDS-gR12-R	AAACtttccggagtagtaccttac	Reverse primer to prepare gRNA

GAAC-ROC5-gR12-F	TGGCacgcaactggagcggcacga	Forward primer to prepare gRNA
GAAC-ROC5-gR12-R	AAACtcgtgccgctccagttgcgt	Reverse primer to prepare gRNA
CAAA-PDS-gR13-F	tggcATGAGTAGGAAGTTGTCCCG	Forward primer to prepare gRNA
CAAA-PDS-gR13-R	aaacCGGGACAACTTCCTACTCAT	Reverse primer to prepare gRNA
CAAA-ROC5-gR13-F	GTGTgcaagccaacgcctggcgc	Forward primer to prepare gRNA
CAAA-ROC5-gR13-R	AAACgcgccaggcgttggcttgc	Reverse primer to prepare gRNA
CAAT-PDS-gR14-F	TGGCacCTGAAGAAATCGGTTTAAAG	Forward primer to prepare gRNA
CAAT-PDS-gR14-R	aaacCTTTAAACCGATTTCTTCAGgt	Reverse primer to prepare gRNA
CAAT-ROC5-gR14-F	TGGCActcaagcactgttgccttag	Forward primer to prepare gRNA
CAAT-ROC5-gR14-R	AAACctaaggcaacagtgcttgagT	Reverse primer to prepare gRNA
CAAG-PDS-gR15-F	tggcaTTTTCCTTTATTCCTTAAGA	Forward primer to prepare gRNA
CAAG-PDS-gR15-R	aaacTCTTAAGGAATAAAGGAAAAt	Reverse primer to prepare gRNA
CAAG-ROC5-gR15-F	GTGTgcggcacgagaacgcgcttct	Forward primer to prepare gRNA
CAAG-ROC5-gR15-R	AAACagaagcgcgttctcgtgccgc	Reverse primer to prepare gRNA
CAAC-PDS-gR16-F	tggcAAGCAAGATCTTTTGCGGGA	Forward primer to prepare gRNA
CAAC-PDS-gR16-R	aaacTCCCGCAAAAGATCTTGCTT	Reverse primer to prepare gRNA
CAAC-ROC5-gR16-F	TGGCatcttcgttttaccaagacg	Forward primer to prepare gRNA
CAAC-ROC5-gR16-R	AAACcgtcttggtaaaacgaagat	Reverse primer to prepare gRNA
SM-21-RFLP-F3	cggcatcagctctcagtcac	For testing genome editing activity at SM21 site
SM-21-RFLP-R3	gatgcacacttctctcacg	For testing genome editing activity at SM21 site
SM-15-RFLP-F	CTCCAGCTTCTTGAAATAAG	For testing genome editing activity at SM15 site
SM-15-RFLP-R	GAGTAGTACCTTACGTATTA	For testing genome editing activity at SM15 site
SM-13-RFLP-F	GACCACGATGTGACTGCTATC	For testing genome editing activity at SM13 and SM17 sites
SM-13-RFLP-R	GAGTTGCTTCAGCATGGATAC	For testing genome editing activity at SM13 and SM17 sites
SM1-RFLP-F	GCTCCAGCAATCACGACCTG	For testing genome editing activity at SM1, SM25, SM31, and SM5 sites
SM1-RFLP-R	CTGATACAGTGATACTTGGC	For testing genome editing activity at SM1, SM25, SM31, and SM5 sites
SM23-RFLP-F	GAGTTCCATCAGTAAGTGC	For testing genome editing activity at SM23, SM11, and SM27 sites
SM23-RFLP-R	GTGTTCCTGATCGAGTGAAC	For testing genome editing activity at SM23, SM11, and SM27 sites
SM3-RFLP-F	GACTATGTATGAACAGGAG	For testing genome editing activity at SM3 site
SM3-RFLP-R	GCTCATATTCACTGTTAGTAG	For testing genome editing activity at SM3 site
SM32-RFLP-F	CCGTCTTGTGTAGGCTGTTC	For testing genome editing activity at SM32, SM10, SM8, SM2, SM24, SM30, and SM22 sites
SM32-RFLP-R	GTTGAGCTCGTCCTTGAGG	For testing genome editing activity at SM32, SM10, SM8, SM2, SM24, SM30, and SM22 sites
SM16-RFLP-F	CGAACTTGTCAAGATGGCAC	For testing genome editing activity at SM16, SM18, SM28 sites
SM16-RFLP-R	CTACGTCTTACACTATGTAAG	For testing genome editing activity at SM16, SM18, SM28 sites
SM14-RFLP-F	GTGGTCTGACATGTTCTCG	For testing genome editing activity at SM14, SM4, SM20 sites
SM14-RFLP-R	CATAGTGAACCATTATTGTG	For testing genome editing activity at SM14, SM4, SM20 sites
SM6-RFLP-F	GTCAACAGTATAGGTTCAC	For testing genome editing activity at SM6, SM26 sites
SM6-RFLP-R	CACAGTTGATTCCGCAAATTG	For testing genome editing activity at SM6, SM26 sites
SM12-RFLP-F	GTCCTCTGCTACAGTGACAG	For testing genome editing activity at SM12 site
SM12-RFLP-R	CTCCATTGCTGAGGATGTC	For testing genome editing activity at SM12 site
ISM-Ins_F	gtactcgatcggcCTCGCTATTGGGACTAACTC	For preparing pYPQ265H, 266B, and 262m3

ISM-Ins_R	ccgctggccgcttGTCCTCTCCTATTTTTGAGAG	For preparing pYPQ265H, 266B, and 262m3
zISMBE-BB_F	ataggagaggacAAGCGGCCAGCGGCGACGAA	For preparing pYPQ265H, 266B, and 262m3
zISMBE-BB_R	cccaatagcgagGCCGATCGAGTACTTCTTGTCAGCAGCTG	For preparing pYPQ265H, 266B, and 262m3
SM17-NGS-F1	ACAGTGGCTCCAGCAATCACGACCTG	NGS primer for Base Editing with z-iSpyMacas9 BEs
SM17-NGS-R1	CAACTATGGAGTGAAATCTCTTGTC	NGS primer for Base Editing with z-iSpyMacas9 BEs
SM17-NGS-F2	AGTCAAGCTCCAGCAATCACGACCTG	NGS primer for Base Editing with z-iSpyMacas9 BEs
SM17-NGS-R2	CACGATTGGAGTGAAATCTCTTGTC	NGS primer for Base Editing with z-iSpyMacas9 BEs
SM17-NGS-F3	AGTTCCGCTCCAGCAATCACGACCTG	NGS primer for Base Editing with z-iSpyMacas9 BEs
SM17-NGS-R3	CCAACATGGAGTGAAATCTCTTGTC	NGS primer for Base Editing with z-iSpyMacas9 BEs
SM17-NGS-F4	TTAGGCGCTCCAGCAATCACGACCTG	NGS primer for Base Editing with z-iSpyMacas9 BEs
SM17-NGS-R4	CACTCATGGAGTGAAATCTCTTGTC	NGS primer for Base Editing with z-iSpyMacas9 BEs
SM21-NGS-F1	TAGCTTGTGGAGAGTTGGGCACCCAC	NGS primer for Base Editing with z-iSpyMacas9 BEs
SM21-NGS-R1	CAAAAGGTGCTCAGTCTGTAGTGGAG	NGS primer for Base Editing with z-iSpyMacas9 BEs
SM21-NGS-F2	CTTGTAGTGGAGAGTTGGGCACCCAC	NGS primer for Base Editing with z-iSpyMacas9 BEs
SM21-NGS-R2	TATAATGTGCTCAGTCTGTAGTGGAG	NGS primer for Base Editing with z-iSpyMacas9 BEs
SM21-NGS-F3	CCGTCCGTGGAGAGTTGGGCACCCAC	NGS primer for Base Editing with z-iSpyMacas9 BEs
SM21-NGS-R3	TCATTCGTGCTCAGTCTGTAGTGGAG	NGS primer for Base Editing with z-iSpyMacas9 BEs
SM2-NGS-F1	ACAGTGGAATCGCCGCACGCAGATGAAG	For testing genome editing activity at SM2 site
SM2-NGS-R1	CAACTAGCTCCTCTAGGGACACCTCC	For testing genome editing activity at SM2 site
SM6/26-NGS-F1	AGTCATCCTGTAGGTCACATGGGTTG	For testing genome editing activity at SM6 and SM26 sites
SM6/26-NGS-R1	CAGGATCACAGTTGATTCCGCAAATTG	For testing genome editing activity at SM6 and SM26 sites
SM6/26-NGS-F2	AGTTCCCCTGTAGGTCACATGGGTTG	For testing genome editing activity at SM6 and SM26 sites
SM6/26-NGS-R2	CCAACACACAGTTGATTCCGCAAATTG	For testing genome editing activity at SM6 and SM26 sites
SM6/26-NGS-F3	TTAGGCCCTGTAGGTCACATGGGTTG	For testing genome editing activity at SM6 and SM26 sites
SM6/26-NGS-R3	CACTCACACAGTTGATTCCGCAAATTG	For testing genome editing activity at SM6 and SM26 sites
SM6/26-NGS-F4	TAGCTTCCTGTAGGTCACATGGGTTG	For testing genome editing activity at SM6 and SM26 sites
SM6/26-NGS-R4	CAAAAGCACAGTTGATTCCGCAAATTG	For testing genome editing activity at SM6 and SM26 sites
SM10-NGS-F1	CTTGTAGAATCGCCGCACGCAGATGAAG	For testing genome editing activity at SM10 site
SM10-NGS-R1	TATAATGCTCCTCTAGGGACACCTCC	For testing genome editing activity at SM10 site
SM10-NGS-F2	CCGTCCGAATCGCCGCACGCAGATGAAG	For testing genome editing activity at SM10 site
SM10-NGS-R2	TCATTCGCTCCTCTAGGGACACCTCC	For testing genome editing activity at SM10 site
SM10-NGS-F3	ACAGTGGAATCGCCGCACGCAGATGAAG	For testing genome editing activity at SM10 site
SM10-NGS-R3	CAACTAGCTCCTCTAGGGACACCTCC	For testing genome editing activity at SM10 site
SM10-NGS-F4	AGTCAAGAATCGCCGCACGCAGATGAAG	For testing genome editing activity at SM10 site
SM10-NGS-R4	CACGATGCTCCTCTAGGGACACCTCC	For testing genome editing activity at SM10 site
150-R221K-F	cttgagaaccttatcgctcagcttcc	For targeted mutagenesis to make pYPQ150-iSpymac
150-R221K-R	CTtacgagacttagaaagtctagcagaaag	For targeted mutagenesis to make pYPQ150-iSpymac
150-N394K-F	agagaggatcttcttagaaagcagagaa	For targeted mutagenesis to make pYPQ150-iSpymac
150-N394K-R	Cttcaacttaacaagaagctcctcggtt	For targeted mutagenesis to make pYPQ150-iSpymac
166-R221K-F	ctcgagaacctgatcgcccagc	For targeted mutagenesis to make pYPQ166-iSpymac
166-R221K-R	CTTccgagacttcgagagcctagcgg	For targeted mutagenesis to make pYPQ166-iSpymac
166-N394K-F	cgcgaggacctcctgaggaagc	For targeted mutagenesis to make pYPQ166-iSpymac
166-N394K-R	Cttgagcttcaccagcagctcctcc	For targeted mutagenesis to make pYPQ166-iSpymac

Supplemental Table 2. T-DNA expression constructs used in this study.

Name	Targeted rice gene	PAM (5'-->3')	gRNA	gRNA expression type	Purpose	Figure shown
pLR1812	OsPDS	TAAG	OsPDS-gRNA07	pOsU6-gRNA	Testing pco-SpyMacCas9 at targets associated with selected NAAN PAMs	Figure 2B
pLR1813	OsPDS	TAAC	OsPDS-gRNA08	pOsU3-gRNA	Testing pco-SpyMacCas9 at targets associated with selected NAAN PAMs	Figure 2B
pLR1814	OsPDS	GAAA	OsPDS-gRNA09	pOsU6-gRNA	Testing pco-SpyMacCas9 at targets associated with selected NAAN PAMs	Figure 2B
pLR1815	OsPDS	GAAG	OsPDS-gRNA11	pOsU6-gRNA	Testing pco-SpyMacCas9 at targets associated with selected NAAN PAMs	Figure 2B
pLR1817	OsPDS	TAAG	OsPDS-gRNA07	pOsU6-gRNA	Testing z-SpyMacCas9 at targets associated with selected NAAN PAMs	Figure 2B
pLR1818	OsPDS	TAAC	OsPDS-gRNA08	pOsU3-gRNA	Testing z-SpyMacCas9 at targets associated with selected NAAN PAMs	Figure 2B
pLR1819	OsPDS	GAAA	OsPDS-gRNA09	pOsU6-gRNA	Testing z-SpyMacCas9 at targets associated with selected NAAN PAMs	Figure 2B
pLR1820	OsPDS	GAAG	OsPDS-gRNA11	pOsU6-gRNA	Testing z-SpyMacCas9 at targets associated with selected NAAN PAMs	Figure 2B, 3A
pLR1822	OsPDS	TAAG	OsPDS-gRNA07	pOsU6-gRNA	Testing pco-iSpyMacCas9 at targets associated with selected NAAN PAMs	Figure 2B
pLR1823	OsPDS	TAAC	OsPDS-gRNA08	pOsU3-gRNA	Testing pco-iSpyMacCas9 at targets associated with selected NAAN PAMs	Figure 2B
pLR1824	OsPDS	GAAA	OsPDS-gRNA09	pOsU6-gRNA	Testing pco-iSpyMacCas9 at targets associated with selected NAAN PAMs	Figure 2B
pLR1825	OsPDS	GAAG	OsPDS-gRNA11	pOsU6-gRNA	Testing pco-iSpyMacCas9 at targets associated with selected NAAN PAMs	Figure 2B
pLR1827	OsPDS	TAAG	OsPDS-gRNA07	pOsU6-gRNA	Testing z-iSpyMacCas9 at targets associated with NAAN PAMs	Figure 2B, 2C
pLR1828	OsPDS	TAAC	OsPDS-gRNA08	pOsU3-gRNA	Testing z-iSpyMacCas9 at targets associated with NAAN PAMs	Figure 2B, 2C
pLR1829	OsPDS	GAAA	OsPDS-gRNA09	pOsU6-gRNA	Testing z-iSpyMacCas9 at targets associated with NAAN PAMs	Figure 2B, 2C, 3C
pLR1830	OsPDS	GAAG	OsPDS-gRNA11	pOsU6-gRNA	Testing z-iSpyMacCas9 at targets associated with NAAN PAMs	Figure 2B, 2C, 3B
pLR2218	OsPDS	AAAA	OsPDS-gRNA01	pOsU3-gRNA	Testing z-iSpyMacCas9 at targets associated with NAAN PAMs	Figure 2C
pLR2219	OsROC5	AAAA	OsROC5-gRNA01	pOsU6-gRNA	Testing z-iSpyMacCas9 at targets associated with NAAN PAMs	Figure 2C
pLR2220	OsPDS	AAAT	OsPDS-gRNA02	pOsU3-gRNA	Testing z-iSpyMacCas9 at targets associated with NAAN PAMs	Figure 2C
pLR2221	OsROC5	AAAT	OsROC5-gRNA02	pOsU3-gRNA	Testing z-iSpyMacCas9 at targets associated with NAAN PAMs	Figure 2C
pLR2222	OsPDS	AAAG	OsPDS-gRNA03	pOsU3-gRNA	Testing z-iSpyMacCas9 at targets associated with NAAN PAMs	Figure 2C
pLR2223	OsROC5	AAAG	OsROC5-gRNA03	pOsU6-gRNA	Testing z-iSpyMacCas9 at targets associated with NAAN PAMs	Figure 2C, 3D
pLR2224	OsPDS	AAAC	OsPDS-gRNA04	pOsU3-gRNA	Testing z-iSpyMacCas9 at targets associated with NAAN PAMs	Figure 2C
pLR2225	OsROC5	AAAC	OsROC5-gRNA04	pOsU6-gRNA	Testing z-iSpyMacCas9 at targets associated with NAAN PAMs	Figure 2C
pLR2226	OsPDS	TAAA	OsPDS-gRNA05	pOsU3-gRNA	Testing z-iSpyMacCas9 at targets associated with NAAN PAMs	Figure 2C
pLR2227	OsROC5	TAAA	OsROC5-gRNA05	pOsU6-gRNA	Testing z-iSpyMacCas9 at targets associated with NAAN PAMs	Figure 2C
pLR2228	OsPDS	TAAT	OsPDS-gRNA06	pOsU6-gRNA	Testing z-iSpyMacCas9 at targets associated with NAAN PAMs	Figure 2C
pLR2229	OsROC5	TAAT	OsROC5-gRNA06	pOsU3-gRNA	Testing z-iSpyMacCas9 at targets associated with NAAN PAMs	Figure 2C
pLR2230	OsROC5	TAAG	OsROC5-gRNA07	pOsU6-gRNA	Testing z-iSpyMacCas9 at targets associated with NAAN PAMs	Figure 2C
pLR2231	OsROC5	TAAC	OsROC5-gRNA08	pOsU3-gRNA	Testing z-iSpyMacCas9 at targets associated with NAAN PAMs	Figure 2C
pLR2232	OsROC5	GAAA	OsROC5-gRNA09	pOsU3-gRNA	Testing z-iSpyMacCas9 at targets associated with NAAN PAMs	Figure 2C
pLR2233	OsPDS	GAAT	OsPDS-gRNA10	pOsU6-gRNA	Testing z-iSpyMacCas9 at targets associated with NAAN PAMs	Figure 2C
pLR2234	OsROC5	GAAT	OsROC5-gRNA10	pOsU3-gRNA	Testing z-iSpyMacCas9 at targets associated with NAAN PAMs	Figure 2C
pLR2235	OsROC5	GAAG	OsROC5-gRNA11	pOsU6-gRNA	Testing z-iSpyMacCas9 at targets associated with NAAN PAMs	Figure 2C
pLR2236	OsPDS	GAAC	OsPDS-gRNA12	pOsU6-gRNA	Testing z-iSpyMacCas9 at targets associated with NAAN PAMs	Figure 2C
pLR2237	OsROC5	GAAC	OsROC5-gRNA12	pOsU3-gRNA	Testing z-iSpyMacCas9 at targets associated with NAAN PAMs	Figure 2C
pLR2238	OsPDS	CAAA	OsPDS-gRNA13	pOsU3-gRNA	Testing z-iSpyMacCas9 at targets associated with NAAN PAMs	Figure 2C
pLR2239	OsROC5	CAAA	OsROC5-gRNA13	pOsU6-gRNA	Testing z-iSpyMacCas9 at targets associated with NAAN PAMs	Figure 2C
pLR2240	OsPDS	CAAT	OsPDS-gRNA14	pOsU3-gRNA	Testing z-iSpyMacCas9 at targets associated with NAAN PAMs	Figure 2C
pLR2241	OsROC5	CAAT	OsROC5-gRNA14	pOsU3-gRNA	Testing z-iSpyMacCas9 at targets associated with NAAN PAMs	Figure 2C
pLR2242	OsPDS	CAAG	OsPDS-gRNA15	pOsU3-gRNA	Testing z-iSpyMacCas9 at targets associated with NAAN PAMs	Figure 2C
pLR2243	OsROC5	CAAG	OsROC5-gRNA15	pOsU6-gRNA	Testing z-iSpyMacCas9 at targets associated with NAAN PAMs	Figure 2C
pLR2244	OsPDS	CAAC	OsPDS-gRNA16	pOsU3-gRNA	Testing z-iSpyMacCas9 at targets associated with NAAN PAMs	Figure 2C
pLR2245	OsROC5	CAAC	OsROC5-gRNA16	pOsU3-gRNA	Testing z-iSpyMacCas9 at targets associated with NAAN PAMs	Figure 2C
pLR2294	OsPDS	GAAA	OsPDS-gRNA09	pOsU6-gRNA	Testing C to T base editing with hAID-z-iSpyMacCas9-D10A-UGI base editor	Figure 4B, 4E
pLR2295	OsPDS	GAAA	OsPDS-gRNA09	pOsU6-gRNA	Testing C to T base editing with z-iSpyMacCas9-D10A-PmCDA-UGI base editor	Figure 4B, 4E
pLR2300	OsPDS	GAAG	OsPDS-gRNA11	pOsU6-gRNA	Testing A to G base editing with wtTadA-TadA*-z-iSpyMacCas9-D10A base editor	Figure 5B
pLR2764	OsROC5	AAAG	OsROC5-gRNA03	pOsU6-gRNA	Testing C to T base editing with hAID-z-iSpyMacCas9-D10A-UGI base editor	Figure 4B, 4C
pLR2765	OsROC5	AAAG	OsROC5-gRNA03	pOsU6-gRNA	Testing C to T base editing with z-iSpyMacCas9-D10A-PmCDA-UGI base editor	Figure 4B, 4C
pLR2767	OsROC5	AAAG	OsROC5-gRNA03	pOsU6-gRNA	Testing A to G base editing with wtTadA-TadA*-z-iSpyMacCas9-D10A base editor	Figure 5B
pLR2768	OsROC5	TAAA	OsROC5-gRNA05	pOsU6-gRNA	Testing C to T base editing with hAID-z-iSpyMacCas9-D10A-UGI base editor	Figure 4B, 4D
pLR2769	OsROC5	TAAA	OsROC5-gRNA05	pOsU6-gRNA	Testing C to T base editing with z-iSpyMacCas9-D10A-PmCDA-UGI base editor	Figure 4B, 4D, 4G, Supp. Fig. 1
pLR2771	OsROC5	TAAA	OsROC5-gRNA05	pOsU6-gRNA	Testing A to G base editing with wtTadA-TadA*-z-iSpyMacCas9-D10A base editor	Figure 5B, 5C
pLR2772	OsROC5	CAAA	OsROC5-gRNA13	pOsU6-gRNA	Testing C to T base editing with hAID-z-iSpyMacCas9-D10A-UGI base editor	Figure 4B, 4F
pLR2773	OsROC5	CAAA	OsROC5-gRNA13	pOsU6-gRNA	Testing C to T base editing with z-iSpyMacCas9-D10A-PmCDA-UGI base editor	Figure 4B, 4F, 4H, Supp. Fig. 2
pLR2775	OsROC5	CAAA	OsROC5-gRNA13	pOsU6-gRNA	Testing A to G base editing with wtTadA-TadA*-z-iSpyMacCas9-D10A base editor	Figure 5B

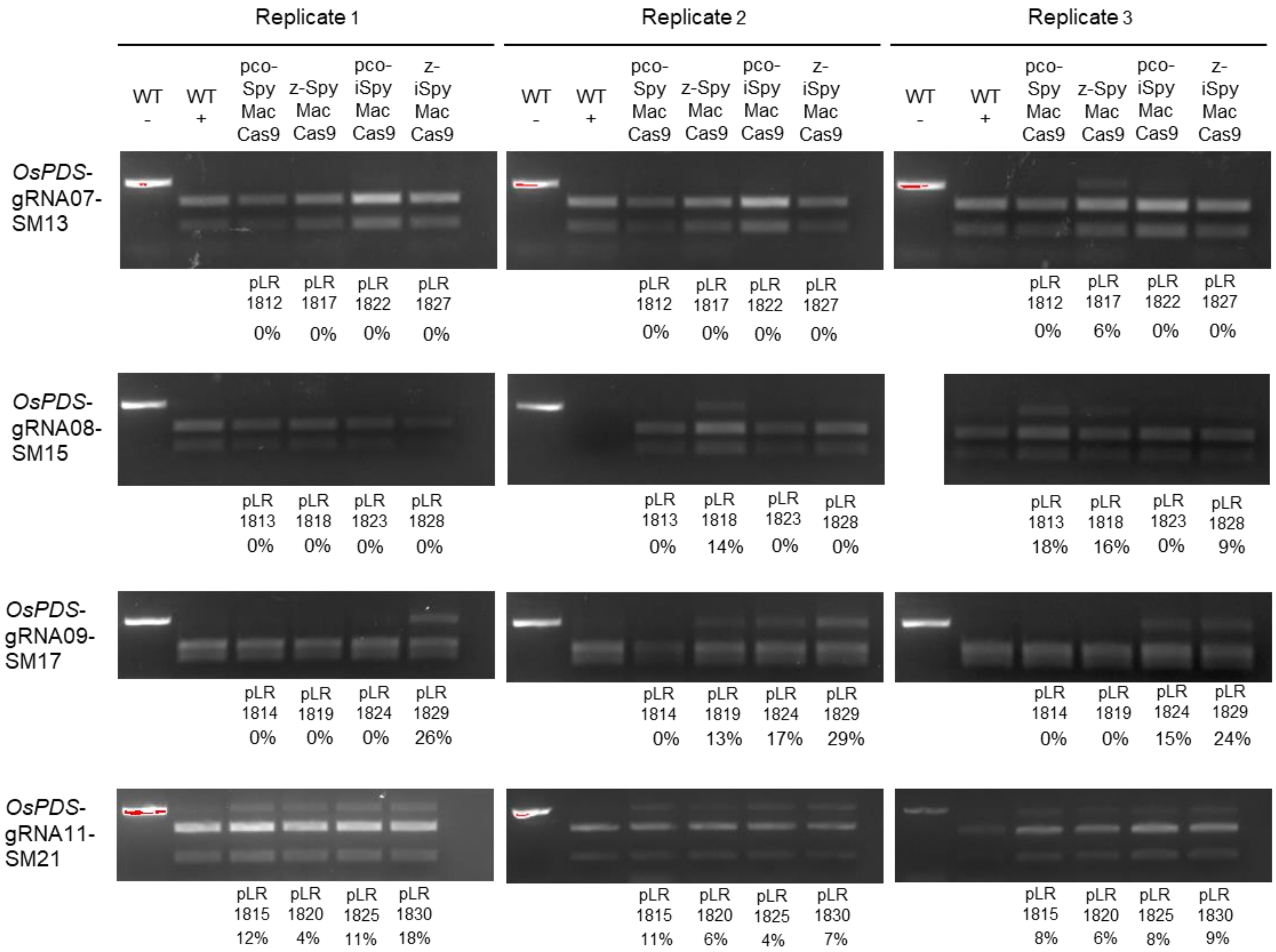

Supplemental Figure 1. Raw RFLP data of testing 4 (i)SpyMacCas9 variants at 4 target sites in 3 replicates.

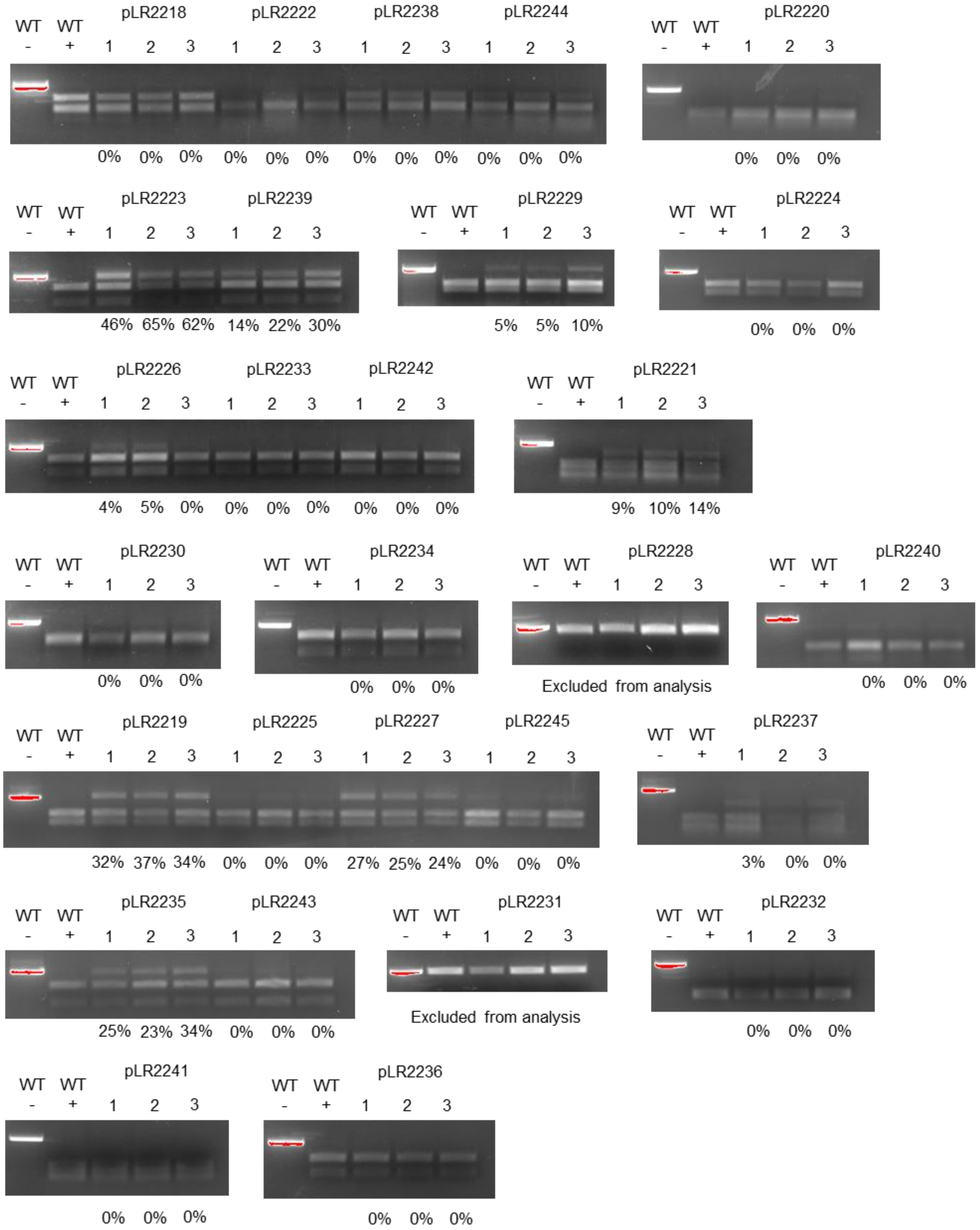

Supplemental Figure 2: Raw RFLP data of testing z-iSpyMacCas9 at additional 28 target sites in 3 replicates. In total, the nuclease activity of z-iSpyMacCas9 was screened 32 target sites. Data from two target sites were excluded from analysis due to enzyme inactivity.

```
pLR2769:     z-iSpyMacCas9-PmCDA1-UGI + OsROC5-sgRNA05-SM10
             AGCGCGTTCTCGTGCCGCTCCAGTTGCGTCTTGGTAAAACGAAGATCGCGGC   WT(OsPDS)
pLR2769-7
Allele 1:    AGCGCGTT-----------------------TGGTAAAACGAAGATCGCGGC   -23bp
Allele 2:    AGCGCGTTCT(64-bp del)CGTGTCAGGAAATTGCACGAAACGAA        -64bp
pLR2769-13
Allele 1:    AGCGCGTTTCTCGTGCCGCTCCAGTTGCGTCTTGGTAAAACGAAGATCGCGGC  +1bp
Allele 2:    AGCGCGTTCCTCGTGCCGCTCCAGTTGCGTCTTGGTAAAACGAAGATCGCGGC  +1bp
```

Supplemental Figure 3. T0 indel mutants generated by PmCDA1 based iSpyMacCas9 CBE with OsROC5-sgRNA05. The PAM is highlighted in red and the protospacer is highlighted in blue.

pLR2773:	z-iSpyMacCas9-PmCDA1-UGI + *OsROC5*-sgRNA13-SM26	
	TGGCGCTGCAGCGTCGCAAGCCAACGCCTGGCGCCAAAGGCGAGACCAGA	WT(OsPDS)
pLR2773-2		
Allele 1:	TGGCGCTGCAGCGTCG-AAGCCAACGCCTGGCGCCAAAGGCGAGACCAGA	-1bp
Allele 2:	TGGCGCTGCAGCGTCGTAAGCCAACGCCTGGCGCCAAAGGCGAGACCAGA	C2T
pLR2773-8		
Allele 1:	TGGCGCTGCAGCGTCGCAAGCCAACGCCTGGCGCCAAAGGCGAGACCAGA	WT
Allele 2:	TGGCGCTGCAGCGAAGTCAACC----CCTGGCGCCAAAGGCGAGACCAGA	T-2A; C-2A; C2T; A3C; G5A; -4bp
pLR2773-20		
Allele 1:	TGGCGCTGCAGCGTCGCAAGCCAACGCCTGGCGCCAAAGGCGAGACCAGA	WT
Allele 2:	TGGCGCTGCAGCGTCGGAAGCCAAC-----GCGCCAAAGGCGAGACCAGA	C2G; -5bp
pLR2773-22		
Allele 1:	TGGCGCTGCAG---------------------------------ACCAGA	-33bp
Allele 2:	TGGCGCTGCAGCGTCGGAAGCCAACGCCTGGCGCCAAAGGCGAGACCAGA	C2G
pLR2773-25		
Allele 1:	TGGCGCTGCAGCGTCGCAAGC---------CAACGCCTGGCGCCAAAGGC	WT
Allele 2:	TGGCGCTGCAGCGTCGCAAGCGTCCTAAGCCAACGCCTGGCGCCAAAGGC	+9bp

Supplemental Figure 4. Additional mutated T0 lines induced by PmCDA1 based iSpyMacCas9 CBE with OsROC5-sgRNA13. The PAM is highlighted in red and the protospacer is highlighted in blue.
